# Supplementary material for: Integrating functional connectivity in designing networks of protected areas under climate change: A caribou case-study
Source: PLoS One. 2020 Sep 30;15(9):e0238821. doi: 10.1371/journal.pone.0238821 (PMC7526922; doi:10.1371/journal.pone.0238821)
Supplement: S1 Material — (DOCX) [file pone.0238821.s001.docx]

**Integrating functional connectivity in designing networks of protected areas under climate change: a caribou case-study**

Sarah Bauduin, Steven G. Cumming, Martin-Hugues St-Laurent and Eliot J.B. McIntire

**S1 Supporting Information.** Construction of the potential future landscapes.

This document explains the construction of the potential future landscapes we used with the spatially explicit individual-based model (Bauduin et al. 2016) according to the four climate change scenarios.

We evaluated four different climate change scenarios. CC_0_ was a scenario without climate change while scenarios CC_Min_, CC_Med_ and CC_High_ represented a gradient of minimum, medium and high climate change impacts. These scenarios represented possible climate change impacts. They did not correspond to any particular climate forecasts such as those of the Intergovernmental Panel on Climate Change (IPCC) assessment reports (e.g., IPCC 2007) as it was beyond the scope of this study to do detailed simulations of the vegetation under the different IPCC scenarios. Climate impact scenarios were defined in terms of their effects on vegetation. The effects were modelled by changing the rules for vegetation succession and disturbances based on the literature. We applied these rules to the current landscape to forecast the potential state in 2080 of alpine tundra, mature fir stands (older than 50 years) and regenerating stands (younger than 30 years) for each climate scenario. These three habitats were the ones used to generate the habitat quality maps (Gaudry 2013) to be used with the spatially explicit individual-based model (Bauduin et al. 2016) to simulate caribou movement. These three habitats were modeled because of their (positive or negative) impact on the caribou (Gaudry 2013). All the other types of habitats (e.g., young fir stands, mature non-fir stands, agricultural fields, etc.) were not predicted as they were not included in the model to generate the habitat quality maps. The year 2080 was chosen because of the data availability for vegetation and disturbances, which are rarely forecast further than the time period 2070-2100 (Gray 2008; Régnière et al. 2012; Logan 2012; Périé et al. 2014). Even though forests are slow-changing systems and drastic changes in composition are unlikely to happen over the next 60 years, we defined our time horizon as 2080 to be cautious regarding the data availability and knowledge of the systems.

*Alpine tundra*

Alpine tundra in Gaspésie is climate driven (Dumais et al. 2014) with wind being a major factor (Renard et al. 2015). Even though no change was observed in the treeline position between 1975 and 2008, researchers noticed a shrub densification of American dwarf birch (*Betula glandulosa*) above the treeline and a development of a more erected tree form for some white spruce (*Picea glauca*) usually found as krummholz (Dumais et al. 2014). *B. glandulosa* radial growth is positively associated with summer temperatures (Dumais et al. 2014) and climate models predicted an increase of these temperatures for the Gaspésie peninsula by 2090 (Logan 2012). It is therefore possible that alpine tundra may be colonized by upright vegetation (i.e., erect trees) on its rim due to climate change. The tundra was assumed constant under CC_0_. We shrunk the tundra polygons (as defined in Gaudry 2013) using interior buffering in the scenarios including climate change. No estimation of alpine tundra reduction was available from the literature or experts, so shrinkage amounts were chosen for simplicity and to show significant difference with the current state. For most tundra sites, we buffered by 100, 200 and 500m in CC_Min_, CC_Med_ and CC_High_, respectively. The exception was the Mount Albert summit, the plateau in the center of the Gaspésie National Park (Mosnier et al. 2003). Mount Albert is composed of serpentine soils (Sirois and Grandtner 1992) which are less subject to vegetation colonization relative to the other tundra areas. We buffered this area by 50 m, 100 m and 250 m for CC_Min_, CC_Med_ and CC_High_, respectively.

*Mature fir stands and regenerating stands*

The abundance and distribution of mature fir stands and regenerating stands are driven by both climate and disturbances.

*Impact of climate change*

Due to climate change, the potential habitat of balsam fir is likely to decrease across Québec (Périé et al. 2014). Predictions of these changes to 2080 have been mapped over eastern North America on a grid cell of 20 x 20 km (source: Ministère des Forêts, de la Faune et des Parcs du Québec, hereafter referred to as MFFP). Potential habitat for balsam fir is predicted to be either lost, lower in quality compared to the present or else mostly unchanged. Potential habitat is predicted to increase in quality in some locations, but not in our study area. Using a GIS, we determined the predicted habitat change at the centroid of each fir stand polygon in our study area. We applied a mortality probability which changed the fir stand into “other” when balsam fir potential habitat was predicted to be either lost or to decline in quality. No mortality probability values were available from the literature or experts so we chose values which provided a reasonable range of consequences among our scenarios. No mortality was applied on fir stands for CC_0_. Mortality probabilities for fir stands where potential habitat conditions were predicted to be lost in 2080 were set at 0.01, 0.10 and 0.50 in CC_Min_, CC_Med_ and CC_High_ respectively. Mortality probabilities in stands where habitat quality decreases were predicted were set at 0.01 and 0.10 in CC_Med_ and CC_High_.

*Impact of disturbances inside protected areas*

Forest harvesting is excluded from protected areas so only rules about natural disturbances were applied to forest stands inside protected areas; climate change impact on fir stands was accounted for beforehand. The dominant natural disturbances in Gaspésie are spruce budworm outbreaks (Saucier et al. 2003) and, to a lesser extent, windthrow. Spruce budworm outbreaks have a mean interval frequency of about 40 years in eastern Québec and this frequency has not changed much since the mid-16^th^ century (Boulanger and Arseneault 2004). Our scenarios were evaluated at 2080 so we defined 2000 as the reference year for spruce budworm impacts in the outbreak cycle, corresponding to two outbreak intervals. Analysis of the maps from the 3^rd^ and 4^th^ forest inventories (source: MFFP), spanning more than two decades, indicated that the impact of windthrow, fire and other disturbances were small compared to that of spruce budworm in our study area. We assumed the magnitude and effects of natural disturbances other than spruce budworm outbreaks constant over the simulation interval. Changes in fire from drought or increased evapotranspiration, as well as changes in windthrow from modifications in ice formation can happen but we do not have clear support to include these elements. Therefore we used 2000 as the reference year for all natural disturbances.

For scenario CC_0_, we assumed that the forest is in a dynamic equilibrium and so the forest age composition in 2000 inside the protected areas resulting from natural disturbances was used to represent that of 2080. Using the ecoforestry maps, we selected all forest polygons inside the protected areas which were undisturbed by human activities prior to 2000. The age of these stands in 2000 was kept to represent their age in 2080. The few stands inside protected areas affected by human disturbances before 2000 were classed as mature in 2080.

Under climate change, spruce budworm outbreak cycles in Gaspésie are predicted to be 10-14 years longer and 26-75% less severe over 2080-2100 than at present (Gray 2008). Spruce budworm population growth rates are predicted to decline during 2041-2070, under expected condition of climate and forest cover (Régnière et al. 2012). Accordingly, we assumed that spruce budworm impacts on forest stands would be less than currently under our climate change scenarios. We simulated this by decreasing the proportion of regenerating stands and therefore increasing the proportion of mature stands as a consequence, in the projected landscapes to reflect a reduction of the mortality due to spruce budworm outbreaks. From the landscape created for CC_0_, we selected the regenerating stands inside protected areas for which spruce budworm outbreak was the recorded disturbance. We randomly sampled some of these forest stands and reclassified them as mature in 2080. Based on Gray (2008), the proportions of stands sampled were 0.25, 0.50 and 0.75 for scenarios CC_Min_, CC_Med_ and CC_High_, respectively.

*Impact of disturbances outside protected areas*

Outside protected areas, the majority of the landscape is managed for timber production (source: Bureau du Forestier en Chef, hereafter BFEC). Efforts are made to prevent or combat spruce budworm outbreaks (Bureau du forestier en chef 2013). Damaged wood is salvaged and plantations may be established in affected areas. Losses to budworm defoliation are accounted for in periodic calculations of annual allowable cut (hereafter referred to as AAC; Bureau du forestier en chef 2013). Windthrow is similarly managed (Bureau du forestier en chef 2013). Therefore, outside protected areas, modifications on forest stands could be regarded as mainly due to forest management. We used the forecasts made by the BFEC to represent the forest composition outside protected areas in 2080 and we did not simulate any extra natural disturbances on these stands.

In Québec, public forest lands are spatially stratified into management units. The BFEC develops management plans and calculates AAC for each unit. There are five management units in Gaspésie, covering 72% of the forest outside protected areas. We assigned the small areas of private forests to these management units, based on the stand proximity with each unit. We applied the BFEC plans to these slightly modified units.

Due to ecosystem management practices in Québec, BFEC plans are expected to increase the amount of old forest and slightly decrease the proportion of regenerating forest relative to the present day. Plans also entail a decreased proportion of fir stands in our study area (source: BFEC). We used the per-unit harvest rates and AACs under all scenarios; climate change impact on fir stands was accounted for beforehand. In each unit, we calculated the proportional decreases in the areas of fir and regenerating stands and increases in old forests from 2008 to 2083. These years were the closest matched to the dates of the ecoforestry maps (2005) and the simulation endpoint (2080). Within each unit, we randomly selected fir and regenerating stands up to the indicated proportional area and reclassified them as follows. Fir stands were reclassified as type “other” for their forest type. Regenerating stands were reclassified as “other” for their age category. The BFEC defined regenerating forest stands as those less than 10 years old (Bureau du forestier en chef 2013). We assumed the indicated proportional reductions applied also to our broader definition of regenerating stands, as those younger than 30 years (Gaudry 2013). The BFEC defined old forest as those older than 80 years (Bureau du forestier en chef 2013), whereas we needed to forecast the abundance of mature forest older than 50 years (Gaudry 2013). In each unit, the projected increase in the abundance of old forest exceeded the remaining area of age between 30 and 50 years or undetermined. Accordingly, in simulated landscapes of 2080, all forest stands not explicitly classed as regenerating were classed as mature. This approximation will not affect scenario outputs as the proportion of forests between 30 and 50 years old are expected to be small compared to the other age categories, especially given ecosystem management practices intended to increase the amount of mature forests.

Once the landscapes were built with the different rules of climate change, we estimated the evolution of the three habitats types we were interested in (i.e., alpine tundra, mature fir stands and regenerating stands). The reference point for the area of each habitat type was the current state as estimated using the 4^th^ forest inventories (source: MFFP). The decrease or increase of the habitat areas were not forced values but the results of the different rules previously described applied to the landscape. For the four climate change scenarios (CC_0_, CC_Min_, CC_Med_ and CC_High_), the alpine tundra area was 100%, 59%, 39%, and 14%, respectively, compared to current conditions. The decrease of the tundra was only driven by climate change. The area of mature fir stands was 107%, 107%, 105%, and 91%, respectively, compared to current conditions. Old stands area increased due to the ecosystem management for the forestry activities. Fir stands area increased due to the reduction of the severity of spruce budworm outbreaks but they also decrease as a consequence of the AAC planned over time and climate change which decreased the habitat conditions. The area of regenerating stands was 68%, 68%, 68%, and 67%, respectively, compared to current conditions.. Regenerating stands area decreased due to the ecosystem management for the forestry activities and the reduction of the severity of spruce budworm outbreaks.

**Literature cited**

Bauduin S, McIntire EJB, St-Laurent M-H, Cumming SG (2016) Overcoming challenges of sparse telemetry data to estimate caribou movement. Ecol Modell 335:24–34. doi: 10.1016/j.ecolmodel.2016.05.004

Boulanger Y, Arseneault D (2004) Spruce budworm outbreaks in eastern Quebec over the last 450 years. Can J For Res 34:1035–1043. doi: 10.1139/X03-269

Bureau du forestier en chef (2013) Manuel de détermination des possibilités forestières 2013-2018. Roberval, QC

Dumais C, Ropars P, Denis M-P, et al (2014) Are low altitude alpine tundra ecosystems under threat? A case study from the Parc National de la Gaspésie, Québec. Environ Res Lett 9:94001. doi: 10.1088/1748-9326/9/9/094001

Gaudry W (2013) Impact des structures anthropiques linéaires sur la sélection d’habitat du caribou, de l’ours noir et du coyote en Gaspésie. Université du Québec à Rimouski

Gray DR (2008) The relationship between climate and outbreak characteristics of the spruce budworm in eastern Canada. Clim Change 87:361–383. doi: 10.1007/s10584-007-9317-5

IPCC (2007) Fourth Assessment Report (AR4) of the United Nations Intergovernmental Panel on Climate Change. Cambridge, UK; New-York, USA

Logan T (2012) Scénarios climatiques pour les régions naturelles de la péninsule de la Gaspésie et la dépression de La Tuque. Québec, QC

Mosnier A, Ouellet J, Sirois L, Fournier N (2003) Habitat selection and home-range dynamics of the Gaspé caribou: a hierarchical analysis. Can J Zool 81:1174–1184. doi: 10.1139/Z03-065

Périé C, de Blois S, Lambert M-C, Casajus N (2014) Effets anticipés des changements climatiques sur l’habitat des espèces arborescentes au Québec. Québec, QC

Régnière J, St-Amant R, Duval P (2012) Predicting insect distributions under climate change from physiological responses: spruce budworm as an example. Biol Invasions 14:1571–1586. doi: 10.1007/s10530-010-9918-1

Renard S, Isabel C, McIntire EJB (2015) Le vent, un facteur déterminant pour le maintien de la toundra alpine. Bull. Conserv. 27–30.

Saucier J-P, Grondin P, Robitaille A, Bergeron J-F (2003) Zones de végétation et les domaines bioclimatiques du Québec.

Sirois L, Grandtner MM (1992) A phyto-ecological investigation of the Mount Albert serpentine plateau. In: The ecology of areas with serpentinized rocks. Springer Netherlands, pp 115–133.
