# Supplementary material for: Integrating functional connectivity in designing networks of protected areas under climate change: A caribou case-study
Source: PLoS One. 2020 Sep 30;15(9):e0238821. doi: 10.1371/journal.pone.0238821 (PMC7526922; doi:10.1371/journal.pone.0238821)
Supplement: S2 Material — (DOCX) [file pone.0238821.s002.docx]

**Integrating functional connectivity in designing networks of protected areas under climate change: a caribou case-study**

Sarah Bauduin, Steven G. Cumming, Martin-Hugues St-Laurent and Eliot J.B. McIntire

**S2 Supporting Information.** Caribou movements in the current and future landscapes.

Maps of the summed number of movements in 1-ha cells done by the simulated caribou over the 10,000 replicates of 4-year model simulations. Figure S2.1 represents the summed movement of the individuals over the current landscape. Figure S2.2 represents the mean of the four resulting summed movement maps done with each of the four climate change landscape scenarios. White areas represent cells never reached by any simulated individuals. The Gaspesie study areas as well as the existing protected areas were overlaid over the color scale representing the movement numbers.


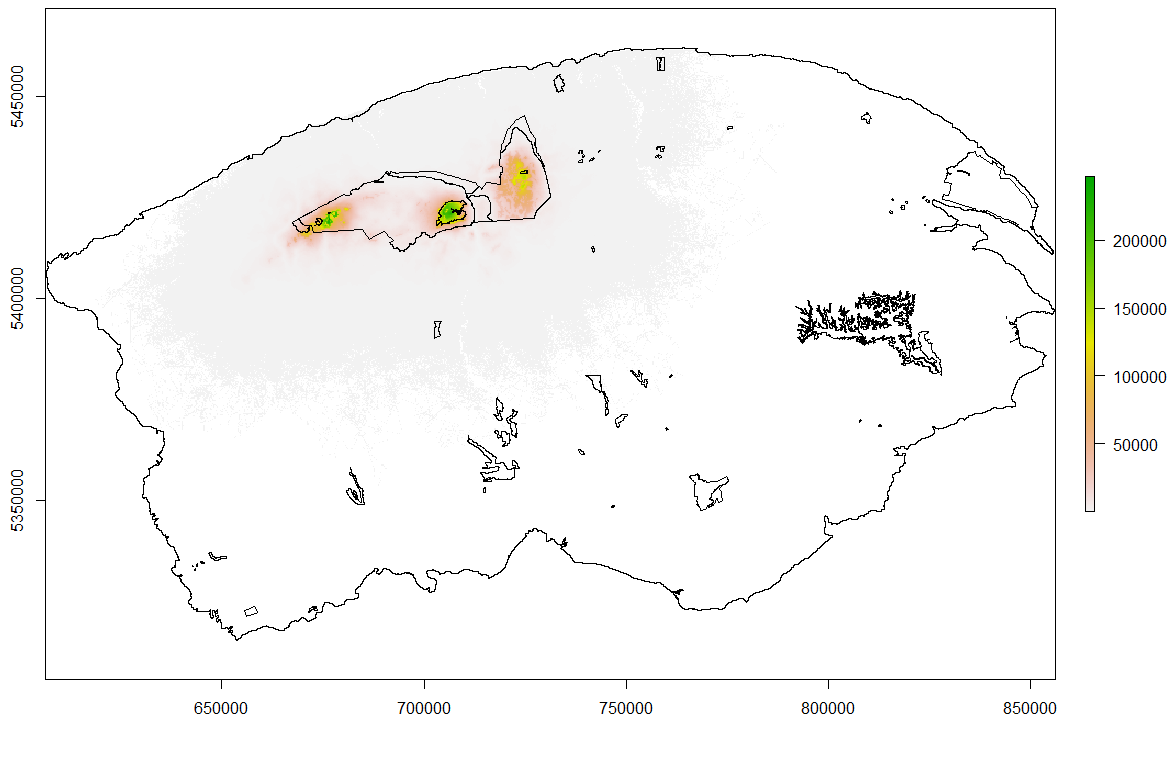


**Figure S2.1: Mean number of caribou movements per cell in the current landscape.**


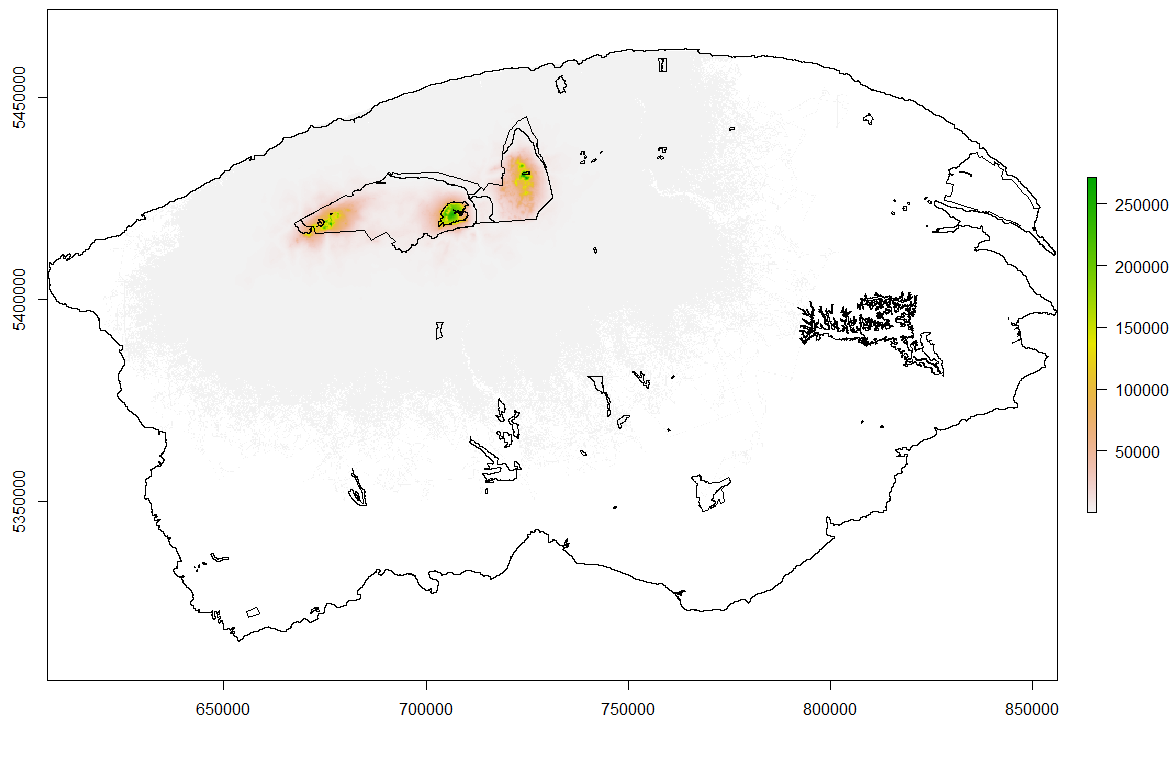


**Figure S2.2: Mean number of caribou movements per cell in the future landscape.**
