# Supplementary material for: Integrating functional connectivity in designing networks of protected areas under climate change: A caribou case-study
Source: PLoS One. 2020 Sep 30;15(9):e0238821. doi: 10.1371/journal.pone.0238821 (PMC7526922; doi:10.1371/journal.pone.0238821)
Supplement: S3 Material — (DOCX) [file pone.0238821.s003.docx]

**Integrating functional connectivity in designing networks of protected areas under climate change: a caribou case-study**

Sarah Bauduin, Steven G. Cumming, Martin-Hugues St-Laurent and Eliot J.B. McIntire

**S3 Supporting Information: Identifying priority conservation areas.**

Here, we present the priority assessments of candidate protected areas under two alternate design criteria, the first aimed at maximizing network ecological representativeness, and the second aimed at maximizing network functional connectivity. The same random sample of networks was used in all three priority assessments.

A quantile value of Q = 0.9989 identified a sample of the best 539 networks in respect to representativeness, which had scores above 6.897 (the selection of a sample slightly larger than the target of 500 networks results essentially from rounding error). The sample networks included 678/690 candidate protected areas. These 678 CPAs are shown (Fig S3.1) color-coded by selection frequency, from low (light orange) to high (dark orange). The inflection point of the rank order curve (not shown) occurred at (31,43). The 31/678 candidate protected areas included in more than 43/539 networks are outlined (see thick lines, Fig S3.1). The existing protected areas are shown in black.


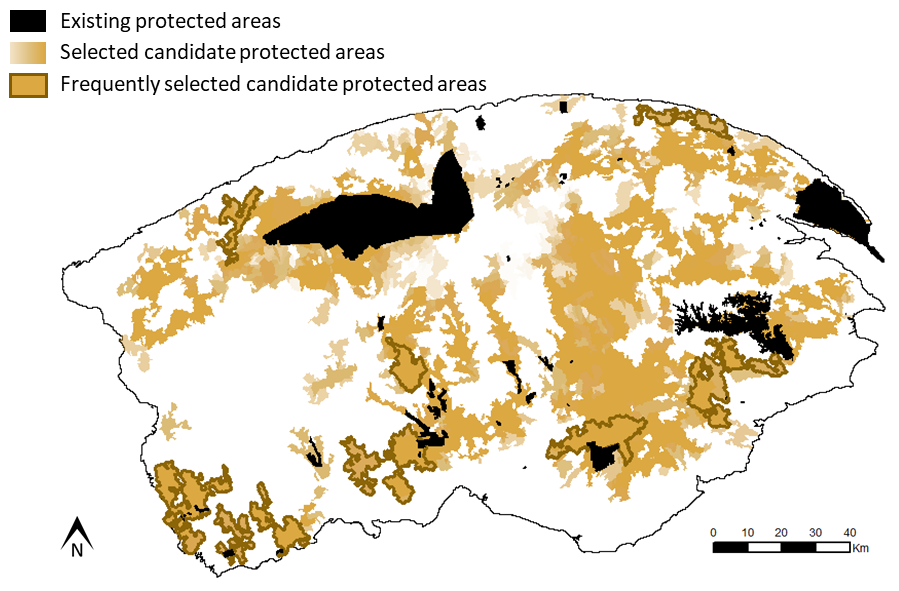


**Figure S3.1: Optimal protected areas networks based on representativeness.**

A quantile value of Q = 0.9988 identified a sample of the best 503 networks in respect to current and future functional connectivity. All current connectivity scores exceeded 467,997. All future connectivity scores exceed 488,139. The sample networks included all 690 candidate protected areas. These CPAs are shown (Fig S3.2) color-coded by selection frequency, from low (light orange) to high (dark orange). The inflection point of the rank order curve (not shown) occurred at 31,43. The 31/690 candidate protected areas included in more than 43/503 networks are outlined (see thick lines, Fig S3.2). The existing protected areas are shown in black.


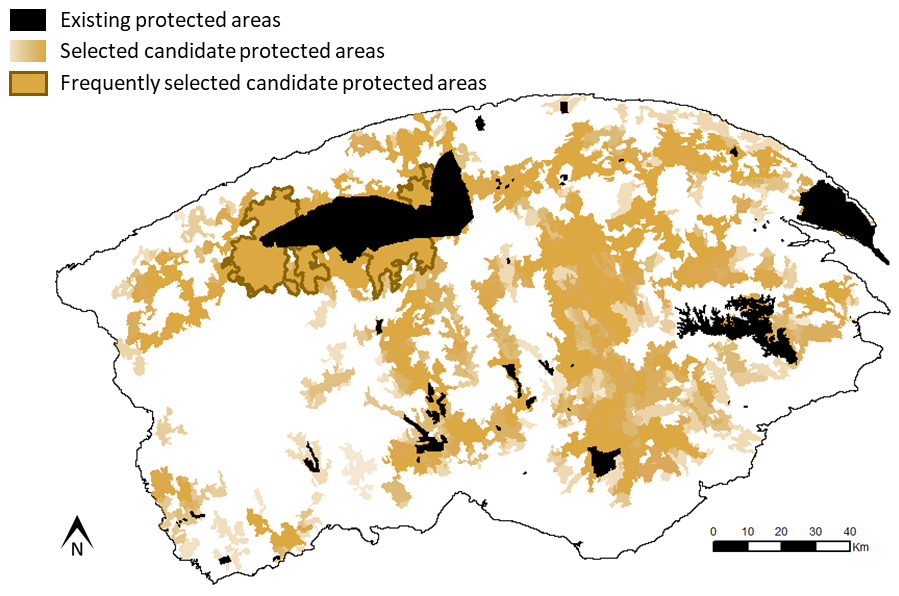


**Figure S3.2: Optimal protected areas networks based on functional connectivity.**
