# Supplementary material for: Integrating functional connectivity in designing networks of protected areas under climate change: A caribou case-study
Source: PLoS One. 2020 Sep 30;15(9):e0238821. doi: 10.1371/journal.pone.0238821 (PMC7526922; doi:10.1371/journal.pone.0238821)
Supplement: Attachment — Submitted filename: PONE-D-20-04608_review.pdf [file pone.0238821.s004.pdf]

PLOS ONE

Coping with climate change when designing networks of protected areas by accounting for functional connectivity: a case-study with caribou

--Manuscript Draft--

**Manuscript Number:**	PONE-D-20-04608
**Article Type:**	Research Article
**Full Title:**	Coping with climate change when designing networks of protected areas by accounting for functional connectivity: a case-study with caribou
**Short Title:**	Climate change, functional connectivity and ecological representativeness in protected areas networks
**Corresponding Author:**	Martin-Hugues St-Laurent, PhD<br>Université du Québec à Rimouski<br>Rimouski, Quebec CANADA
**Keywords:**	
**Abstract:**	Context<br><br>Land-use change and climate change are recognized as two main drivers of the current biodiversity decline. Protected areas attempt to secure the landscape from additional land modifications and likely help species cope with climate change impacts. To maximize effectiveness, protected areas need to cover a representative sample of the regional biodiversity and to be functionally connected, facilitating individual movements among protected areas in a network.<br><br>Objectives<br><br>We developed a methodology to define effective protected areas to implement in a regional network using ecological representativeness and functional connectivity as criteria. We illustrated this methodology in the Gaspésie region of Québec, Canada.<br><br>Methods<br><br>We simulated movements for the endangered Atlantic-Gaspésie caribou population (Rangifer tarandus caribou), using an individual-based model, to determine functional connectivity based on this large mammal. We created multiple protected areas network scenarios and evaluated their ecological representativeness and functional connectivity for the current and future conditions. We selected a subset of the most effective network scenarios and extracted the protected areas included in them.<br><br>Results<br><br>There was a trade-off between ecological representativeness and functional connectivity for the created networks. Only a few protected areas among those available were repeatedly chosen in the most effective networks.<br><br>Conclusions<br><br>Protected areas maximizing both ecological representativeness and functional connectivity represented suitable areas to implement in an effective protected areas network. These areas ensured that a representative sample of the regional biodiversity was covered by the network, as well as maximizing the movement over time between and inside the protected areas for the focal population.
**Order of Authors:**	Martin-Hugues St-Laurent, PhD
	Sarah Bauduin
	Steven G. Cumming
	Eliot J.B. McIntire

Powered by Editorial Manager® and ProduXion Manager® from Aries Systems Corporation

Additional Information:	
**Question**	**Response**
**Financial Disclosure**<br><br>Enter a financial disclosure statement that describes the sources of funding for the work included in this submission. Review the submission guidelines for detailed requirements. View published research articles from *PLOS ONE* for specific examples.<br><br>This statement is required for submission and **will appear in the published article** if the submission is accepted. Please make sure it is accurate.<br><br>**Unfunded studies**<br>Enter: *The author(s) received no specific funding for this work.*<br><br>**Funded studies**<br>Enter a statement with the following details:<br>• Initials of the authors who received each award<br>• Grant numbers awarded to each author<br>• The full name of each funder<br>• URL of each funder website<br>• Did the sponsors or funders play any role in the study design, data collection and analysis, decision to publish, or preparation of the manuscript?<br>• **NO** - Include this sentence at the end of your statement: *The funders had no role in study design, data collection and analysis, decision to publish, or preparation of the manuscript.*<br>• **YES** - Specify the role(s) played.<br><br>* typeset	Funding for this project was provided by:<br>- the Québec Fonds Verts (S.C., E.M.) (URL: http://www.environnement.gouv.qc.ca/ministere/fonds-vert/)<br>- the Ouranos Consortium (S.C., E.M.) (URL: https://www.ouranos.ca/)<br>- the Centre d'étude de la forêt (S.B.) (URL: http://www.cef-cfr.ca/)<br>- Natural Sciences and Engineering Research Council Discovery Grants (E.M., S.C.) (URL: https://www.nserc-crsng.gc.ca/NSERC-CRSNG/)<br>- the Canada Research Chair's program (E. M.) (URL: https://www.chairs-chaires.gc.ca)<br>-the French National Research Agency (ANR-16-CE02-0007, S. B.) (URL: https://anr.fr/en/)
**Competing Interests**<br><br>Use the instructions below to enter a competing interest statement for this submission. On behalf of all authors, disclose any competing interests that could be perceived to bias this work—acknowledging all financial support and any other relevant financial or non-financial competing interests.	The authors have declared that no competing interests exist.

This statement **will appear in the published article** if the submission is accepted. Please make sure it is accurate. View published research articles from *PLOS ONE* for specific examples.<br><br>**NO authors have competing interests**<br><br>Enter: *The authors have declared that no competing interests exist.*<br><br>**Authors with competing interests**<br><br>Enter competing interest details beginning with this statement:<br><br>*I have read the journal's policy and the authors of this manuscript have the following competing interests: [insert competing interests here]*<br><br>* typeset	
**Ethics Statement**<br><br>Enter an ethics statement for this submission. This statement is required if the study involved:<br><br>• Human participants<br>• Human specimens or tissue<br>• Vertebrate animals or cephalopods<br>• Vertebrate embryos or tissues<br>• Field research<br><br>Write "N/A" if the submission does not require an ethics statement.<br><br>General guidance is provided below. Consult the submission guidelines for detailed instructions. **Make sure that all information entered here is included in the Methods section of the manuscript.**	N/A<br><br>In the current manuscript, we are using a model that was developed in two other papers (Bauduin et al. 2016 - Ecological Modeling; Bauduin et al. 2018 - Scientific Reports) for which we used telemetry data collected on several individuals. We had an Animal Welfare certificate (#52-13-112) for this model (see the two above-mentioned references).

Powered by Editorial Manager® and ProduXion Manager® from Aries Systems Corporation

**Format for specific study types**<br><br>**Human Subject Research (involving human participants and/or tissue)**<br>• Give the name of the institutional review board or ethics committee that approved the study<br>• Include the approval number and/or a statement indicating approval of this research<br>• Indicate the form of consent obtained (written/oral) or the reason that consent was not obtained (e.g. the data were analyzed anonymously)<br><br>**Animal Research (involving vertebrate animals, embryos or tissues)**<br>• Provide the name of the Institutional Animal Care and Use Committee (IACUC) or other relevant ethics board that reviewed the study protocol, and indicate whether they approved this research or granted a formal waiver of ethical approval<br>• Include an approval number if one was obtained<br>• If the study involved *non-human primates*, add *additional details* about animal welfare and steps taken to ameliorate suffering<br>• If anesthesia, euthanasia, or any kind of animal sacrifice is part of the study, include briefly which substances and/or methods were applied<br><br>**Field Research**<br><br>Include the following details if this study involves the collection of plant, animal, or other materials from a natural setting:<br>• Field permit number<br>• Name of the institution or relevant body that granted permission	
**Data Availability**<br><br>Authors are required to make all data underlying the findings described fully available, without restriction, and from the time of publication. PLOS allows rare exceptions to address legal and ethical concerns. See the PLOS Data Policy and FAQ for detailed information.	No - some restrictions will apply

Powered by Editorial Manager® and ProduXion Manager® from Aries Systems Corporation

A Data Availability Statement describing where the data can be found is required at submission. Your answers to this question constitute the Data Availability Statement and **will be published in the article**, if accepted.<br><br>**Important:** Stating 'data available on request from the author' is not sufficient. If your data are only available upon request, select 'No' for the first question and explain your exceptional situation in the text box.<br><br>Do the authors confirm that all data underlying the findings described in their manuscript are fully available without restriction?	
**Describe where the data may be found in full sentences. If you are copying our sample text, replace any instances of XXX with the appropriate details.**<br><br>• If the data are **held or will be held in a public repository**, include URLs, accession numbers or DOIs. If this information will only be available after acceptance, indicate this by ticking the box below. For example: *All XXX files are available from the XXX database (accession number(s) XXX, XXX.).*<br>• If the data are all contained **within the manuscript and/or Supporting Information files**, enter the following: *All relevant data are within the manuscript and its Supporting Information files.*<br>• If neither of these applies but you are able to provide **details of access elsewhere**, with or without limitations, please do so. For example:<br><br>*Data cannot be shared publicly because of [XXX]. Data are available from the XXX Institutional Data Access / Ethics Committee (contact via XXX) for researchers who meet the criteria for access to confidential data.*<br><br>*The data underlying the results presented in the study are available from (include the name of the third party*	Data cannot be completely shared publicly because of the Endangered Status of the Atlantic-Gaspésie caribou population, so data that could help track and find these animals won't be made available publicly. However, the modeled data we used to quantify the balance between ecological representativeness and functional connectivity will be made available in a public repository (DRYAD) upon acceptance for researchers who meet the criteria for access to confidential data.

and contact information or URL).<br>• This text is appropriate if the data are owned by a third party and authors do not have permission to share the data.<br><br>* typeset	
Additional data availability information:	Tick here if the URLs/accession numbers/DOIs will be available only after acceptance of the manuscript for publication so that we can ensure their inclusion before publication.; Tick here if your circumstances are not covered by the questions above and you need the journal's help to make your data available.

Manuscript
Click here to download Manuscript Bauduin et al_PLoS One_MS.docx

Coping with climate change when designing networks of protected areas by accounting for functional connectivity: a case-study with caribou

Authors: Sarah Bauduin[1, 2] (sarah.bauduin@cefe.cnrs.fr), Steven G. Cumming[1] (stevec@sbf.ulaval.ca), Martin-Hugues St-Laurent[3, *] (martin-hugues_st-laurent@uqar.ca), and Eliot J.B. McIntire[1, 4] (eliot.mcintire@canada.ca)

[1] Laval University, Faculty of Forestry, Geography and Geomatics, Abitibi-Price building. 2405 rue de la Terrasse, Québec, QC, G1V 0A6, Canada

[2] CEFE, CNRS, University of Montpellier, EPHE, University Paul Valéry Montpellier 3, IRD, Montpellier, France

[3] Université du Québec à Rimouski, Département de Biologie, Chimie et Géographie, Center for Northern Studies, Center for Forest Research. 300 allée des Ursulines, Rimouski, QC, G5L 3A1, Canada.

[4] Canadian Forest Service, Natural Resources Canada. 506 Burnside Road West, Victoria, BC, V8Z 1M5, Canada

*__Corresponding author__: Martin-Hugues St-Laurent

Mailing address: Université du Québec à Rimouski, Département de Biologie, Chimie et Géographie, Center for Northern Studies, Center for Forest Research. 300 allée des Ursulines, Rimouski, QC, G5L 3A1, Canada.

Phone number: +1.418.723.1986 #1538

Email address: martin-hugues_st-laurent@uqar.ca

Abstract

Context: Land-use change and climate change are recognized as two main drivers of the current biodiversity decline. Protected areas attempt to secure the landscape from additional land modifications and likely help species cope with climate change impacts. To maximize effectiveness, protected areas need to cover a representative sample of the regional biodiversity and to be functionally connected, facilitating individual movements among protected areas in a network.

Objectives: We developed a methodology to define effective protected areas to implement in a regional network using ecological representativeness and functional connectivity as criteria. We illustrated this methodology in the Gaspésie region of Québec, Canada.

Methods: We simulated movements for the endangered Atlantic-Gaspésie caribou population (*Rangifer tarandus caribou*), using an individual-based model, to determine functional connectivity based on this large mammal. We created multiple protected areas network scenarios and evaluated their ecological representativeness and functional connectivity for the current and future conditions. We selected a subset of the most effective network scenarios and extracted the protected areas included in them.

Results: There was a trade-off between ecological representativeness and functional connectivity for the created networks. Only a few protected areas among those available were repeatedly chosen in the most effective networks.

Conclusions: Protected areas maximizing both ecological representativeness and functional connectivity represented suitable areas to implement in an effective protected areas network. These areas ensured that a representative sample of the regional biodiversity was covered by the

network, as well as maximizing the movement over time between and inside the protected areas for the focal population.

Keywords

Ecological representativeness; functional connectivity; climate change; movement; coarse filter; fine filter; Atlantic-Gaspésie caribou endangered population

Introduction

Habitat change is recognized as the main driver of the current declines of terrestrial species [1–3]. Securing habitats by creating or expanding protected areas networks is part of the solution to the challenge of biodiversity loss [4]. A regional protected areas network could be considered ultimately effective insofar as it can sustain the region's biodiversity into some reasonably foreseeable future. Such effectiveness is not guaranteed [5,6] and could be limited by many factors [7]. We consider three such factors: ecological representativeness [8], functional connectivity among protected areas within the network [9] and resilience to climate change [10].

Ecological representativeness measures the degree to which the various non-anthropogenic habitats within a focal region are available within a protected areas network in proportion to their regional abundance [11,12]. When protected area locations are skewed towards certain types of habitats [13,14], usually for economic or social reasons, ecological representativeness will be low and high valued habitats for some species may be under-represented [8]. Conversely, when ecological representativeness is high, it is reasonable to assume that the habitat requirements for most species will be satisfied within the protected areas network. This assumption is usual in conservation and representativeness is one of the core concepts in systematic conservation planning [12,15].

A high degree of ecological representativeness is a necessary condition for an effective protected areas network, but it is not sufficient. There are species whose requirements are not automatically satisfied by ecological representativeness alone. One example would be wide-ranging species with habitat requirements varying among seasons or life history stages. Functional connectivity is "the degree to which the landscape facilitates or impedes movement among resource patches" [16] or, as here, among protected areas within a protected areas

network. Functional connectivity is species- or population-specific [17,18]. A protected areas network with high functional connectivity facilitates the movement between different protected areas for individuals of a given species, increasing their access to resources and ultimately the rates of recruitment or survivorship [19]. Increasing functional connectivity may thus increase population size and decrease extinction risk for the vulnerable species [19]. These effects would increase the effectiveness of a protected areas network.

Climate change is among the main current drivers of ecosystem change and its negative impacts on biodiversity have increased rapidly over the past century [1,3,20,21]. Climate change disrupts environmental patterns and species' habitats globally [22,23]. As a result, species distributions and individual movement patterns are impacted [24,25]. The effectiveness of a fixed protected areas network designed for current conditions may decrease in the future, if either ecological representativeness or functional connectivity decline. Enhancing or maintaining functional connectivity inside protected areas networks is thus one approach to helping species cope with climate change [10,26]; individuals would be better able to access resources available in distant protected areas, or even to migrate from less to more favorable areas. Because of the "cost of waiting", managers should proactively account for future climate change effects when implementing new protected areas networks [27] or expanding existing networks. One important effect would be a loss of functional connectivity. Therefore, the ability of a protected areas network to sustain functional connectivity under is another dimension of effectiveness.

Many methods exist for designing protected areas networks to achieve ecological representativeness. In the systematic conservation planning literature [12], variations of the site selection problem are posed, where one seeks a subset of available sites that, in aggregate, achieve some measure of ecological representativeness at a near-minimum of total area or cost.

Variations of these approaches exist that can also partially satisfy other conservation objectives such as topological connectivity, where selected sites are spatially aggregated in some degree [e.g. 28].

The aim of our paper is to present a new method to include functional connectivity in the design process, while accounting also for its persistence under climate change. Our method applies individual-based movement models to simulate maps of habitat use by a population, under present conditions and under hypothetical future conditions reflecting climate change scenarios. From these maps, we derive numerical indices of functional connectivity that allow alternate protected areas network designs to be compared. These are coupled with a novel variant of the site-selection algorithm that constructs protected areas networks of specified total area that can achieve a high degree of ecological representativeness while also satisfying secondary constraints on connectivity and intactness; the variant is presented in detail in Saucier et al. [*in review*].

Our goal is to identify protected areas network designs that simultaneously achieve high degrees of ecological representation and functional connectivity under present conditions and future climates. Achieving this is a multi-objective optimization problem [29,30] that does not admit a unique solution. Instead, one can define a tradeoff surface by those points where no single objective can be increased without decreasing at least another. Such points are said to be Pareto optimal; points on the interior of this surface are sub-optimal in that other solutions exist that are better in terms of all the dimensions considered. Our design methodology uses randomization to generate a large sample of feasible solutions for a given protected areas network design problem. Using these samples, we can approximately define the tradeoff-surface

and identify a set of feasible, near-equivalent solutions in the vicinity of any specified point on that surface.

We illustrated our method in the Gaspésie region (Québec, Canada) using the endangered Atlantic-Gaspésie population of woodland caribou (*Rangifer tarandus caribou*) as our focal population. This isolated herd, that has been identified as one of 12 Designatable Caribou Units considered irreplaceable components of Canada's biodiversity [31], is declining since the late 19th century and reached a critical abundance level [32]. To measure functional connectivity with respect to this population, we applied an individual-based model of animal movement previously developed for this population [33]. The government of Québec is currently engaged in expanding the existing network of protected areas in this region to attain a proportional area target of 12% [34]. To place our work in the context of this ongoing conservation planning exercise, we employed a variant of our design methodology that constructs protected areas networks by adding new protected areas to the existing network. We identified areas that were of high relative importance in achieving ecological representation in the Gaspésie region and functional connectivity for the Atlantic-Gaspésie caribou under present conditions and future climates.

Methods

Study area

The Gaspésie natural region (latitude extent: 47.98 to 49.20º N, longitude extent: 64.11 to 67.57º W; Fig. 1) is a physiographically defined area of approximately 25,000 km² at the eastern end of the Gaspésie peninsula, in eastern Québec, Canada [34]. Except for narrow coastal bands, it belongs to the balsam fir - white birch bioclimatic domain [35]. The climate is maritime with

abundant precipitation. Wildfire is infrequent; the main natural disturbance is spruce budworm (*Choristoneura fumiferana*) outbreaks [35]. Approximately 90% of the region is covered by forests and 80% of these are on public lands [34]. Forest harvesting and the associated extensive road network are the main proximate human disturbances to date. Only 34% of the Gaspésie natural region is free from measurable human footprint [34].

An existing protected areas network covers 5.5% (1371 km²) of the region (Fig. 1). This falls short of the target of an ecologically representative 12%, set by the government of Québec [36]. A scenario proposed by the Ministère de l'Environnement et de la Lutte aux Changements Climatiques du Québec (MELCC) defined 20 new protected areas that would increase the percentage of area protected to 12.3% (3080 km²) [34; Fig. 1].

The Gaspésie National Park (802 km²) is currently the largest protected area in the region [37]. This park helps conserve 42 endangered and vulnerable species of plants and animals [34], including the Atlantic-Gaspésie caribou population. Because of its designated status as endangered [38], its capacity to move widely [39] and the vulnerability of its habitat [40], we chose it as our focal population to define functional connectivity. The population is designated because of its small size – currently ~70 individuals [32] – and the observed long-term decline [41]. These caribou rely on alpine tundra [42,43] as a predator-free refuge against coyotes and black bears [44], a habitat possibly threatened by climate change [45,46]. Caribou also use mid-elevation, old fir forests during winter [43,44]. The fir forests around the park are affected by forest harvesting, and also possibly by climate change [47].

#Figure 1 approximately here#

Building protected areas networks

We adapted the BEACONs (Boreal Ecosystems Analysis for Conservation Networks) approach to build protected areas networks [48,49]. This approach uses mapped hydrological catchments, rather than the cells of an arbitrary grid or other tessellation, as the basic spatial units. This allows the design of protected areas that simultaneously achieve terrestrial and hydrological connectivity [50,51]. Although functional connectivity for aquatic species was not a major concern in our study area [34], it is increasingly recognized as a desirable goal in general [52]. Of more importance to the present application is that the construction does impose some limits on the shape and size of protected areas because of the size of catchments and the method of stream network traversal. However, the size of the catchments was small, implying high spatial resolution to the design. Candidate protected areas are assembled as contiguous, hydrologically connected sets of catchments satisfying minimum size and intactness criteria. The construction proceeds from an initial “seed” catchment, by a modified breadth-first traversal of the stream network. Catchments are added as they are encountered, provided they satisfy a catchment-level intactness criteria. The process continues until the target size is achieved, or all pathways are blocked by headwater or non-intact catchments.

We used a custom catchment layer for Gaspésie defined from the 1:50,000 scale National Hydrological Network (Version 14 NRCAN 2011-2014) and the Canada Digital Elevation Model (NRCAN 2001-2012). The catchment’s average size was 2.3 km^2 (SD = 1.3 km^2). We defined catchment intactness using existing 250 x 250m raster maps of the human footprint in Gaspésie [34]. Six disturbance types were defined: forestry activities, roads and trails, agriculture, power-line rights-of-way, urban areas and “other”. We gave each type equal weight.

As only the first two types are widespread in our study area, we are primarily basing intactness on the absence of recent forest harvesting, roads and trails,

We defined a binary 250 x 250m intactness raster, taking values of 0 where no disturbance of any type was present, and 1 otherwise. Catchment-level intactness was calculated as the mean intactness over all raster cells within a catchment, or equivalently, as the proportion of intact cells. We defined our catchment-level intactness criteria as the median catchment intactness over all catchments on public lands, outside of existing protected areas (= 0.026). In order to create feasible designs given land ownership in Gaspésie, we set the intactness of catchments completely overlapping private lands [34] to zero so that they were excluded from the construction.

We prioritize the inclusion of headwater catchments in protected areas, because of their hydrological importance and to reduce the potential for upstream river contamination inside protected areas [53]. Accordingly, we used all intact headwater catchments as seeds. We used the mean size of the new protected areas proposed by the MELCC (Fig. 1; mean = 85.5 km^2, SD = 61. 4 km^2; [34]) as the target size. The construction adds entire catchments one at a time, so the final size may exceed this target. Because the mean catchment area is small (2.3 km^2) relative to the target size, we consider such discrepancies negligible. We then applied the candidate protected area construction to each seed catchment, with target size and catchment intactness criteria as defined above. The procedure returns a list of the catchments selected, their total area, and their area-weighted mean intactness. We did not apply any protected area-level intactness criteria to sample candidate protected areas as only intact catchments were used to construct them. To make our size criteria more comparable to that used by the MELCC [34], we selected

candidate protected areas with a total area above 31.2 km^2, the size of their smallest proposed new protected area.

We used the set of selected candidate protected areas to generate a sample of 500,000 candidate protected areas networks. Each candidate network was constructed by adding a random sequence of candidate protected areass to the existing network, until the summed area of unique catchments exceeded the MELCC area target of 3080 km^2. The candidate protected areas included within a given candidate network may overlap with each other or with elements of the existing network, but this does not affect the candidate network area. It is easy to show that the properties of hydrological connectivity and intactness are conserved by aggregating overlapping candidate protected areas. Thus, the only material consequence of such overlap is that spatially disjunct network elements may exceed the minimum size criteria, and may be fewer than expected based on the number of candidate protected areas that were added. However, other things being equal, larger protected areas are preferred [54], and the number of spatially separated components is not one of our network design criteria, so we do not consider this overlap of relevance to the present study.

Network ecological representativeness

Ecological representativeness is measured at the candidate network level [48], in terms of four environmental attributes: elevation, surficial deposit, drainage class and potential vegetation type [34]. Potential vegetation was defined by the Ministère des Forêts, de la Faune et des Parcs du Québec (MFFP) as the vegetation present on a site or potentially present, in the absence of disturbance [36]. Surficial deposit, drainage class and potential vegetation type had been used for a gap analysis conducted to inform the proposed expansion of the existing protected areas

network [34]. The four attributes define habitats independent of human activities such as harvesting history. All four attributes were available for the entire study area at adequate spatial resolution (source: MELCC). Catchment-level attributes were calculated using standard spatial data operations.

We measure candidate network representativeness using non-parametric, two-sample univariate dissimilarity measures. For each attribute, we obtain its distributions over all catchments within the candidate network, and over all other catchments within the study region. For continuous attributes (e.g., elevation), we calculate a two-sample Kolmogorov-Smirnov statistic. For categorical attributes (e.g. surficial deposit, drainage class, and potential vegetation type), we calculate the Bray-Curtis statistic. These statistics range from 0 to 1, and are 0 only when the two distributions are identical. That is, they measure a candidate network's deviation from perfect representation with respect to an attribute. The univariate statistics for multiple attributes are combined into a univariate distance metric by calculating a Euclidean norm [48]. We took the inverse of this distance metric as the ecological representativeness score for each candidate network, so that a larger score indicates a greater degree of representativeness.

We also calculated the representativeness score of the proposed MELCC network. To do this, the protected areas within the MELCC network were approximated to the resolution of hydrological catchments. Because these catchments were relatively small, we assumed approximation errors to be negligible. We note that this measure of ecological representativeness does not depend on the specific choice of ecological attributes or on the specifics of our design methodology; it can be applied to any existing protected areas network using whatever ecological attributes are or interest.

Network functional connectivity

We defined candidate network functional connectivity for the Atlantic-Gaspésie caribou population by simulating, using a spatially explicit individual-based movement model adapted from Bauduin et al. [33,55]. The original model was re-estimated from newly available GPS locations [39]. Model movement behavior is partially driven by spatial variation in habitats. Following Bauduin et al. [33], we generated five different habitat maps to represent the current landscape and four landscapes for 2080 forecast under different climate change scenarios. The current landscape was derived from the ecoforestry maps from Québec's 4th decennial forest inventory program (source: MFFP). The scenarios represented a range of potential climate change impacts (none, minimum, moderate and high) on vegetation dynamics and natural disturbances. Primary impacts were reduced areas of tundra [45,56] and of fir forest [47], and a decreased severity of spruce budworm outbreaks [57,58] leading to less area with young fir stands. A complete description of the four climate scenarios and of the construction of the corresponding 2080 landscapes is given in Bauduin et al. [55] and in the Supplementary Material S1. A fifth scenario represented current conditions.

For each landscape, we created 20 individuals in each of the three caribou subpopulation (see [33]; current population estimates are 20, 35 and 15, [32]) and ran 10,000 replicates of four-year model simulations. For each scenario and replicate, we calculated and mapped the number of caribou visits per 1 ha landscape cell. For each candidate network, under each scenario, we measured network functional connectivity by taking the mean, over replicates, of the number of caribou visits in cells within the network's protected areas. This represented the simulated movement patterns within and between the protected areas. The differences in results between climate scenarios were relatively small. We considered evaluating differences between them to

be unimportant relative to our core message concerning the inclusion of functional connectivity in a protected areas network design. Accordingly, and because no climate change scenario was defined as more likely than another, we used the mean over the four climate change scenarios to represent functional connectivity under future climate conditions. We calculated the functional connectivity of the MELCC network under current and future conditions, in the same way.

Identifying priority conservation areas

By plotting the distributions of indicators (i.e., ecological representativeness, current and future functional connectivity), the outlines of the tradeoff surface may be visualized. To identify one location on this surface, we incrementally decreased the quantile Q from 1 until at least 500 networks had all three indicators above the corresponding sample quantiles. This identified a subsample of 0.1% of the candidate networks that were highly ranked under all three criteria. This represents a tradeoff which values all three attributes equally. We then extracted the individual candidate protected areas contained within the subsample of candidate networks and calculated the proportional frequency of their inclusion in the subsample. We kept the candidate protected areas the most frequently selected by using as the frequency threshold the ~~inflexion~~ point in the proportional frequency curve when ranking them from most to least selected in the subsamples networks. Then we mapped the candidate protected areas within the study region color-coded by this proportion. Frequently selected candidate protected areas may be interpreted as suggesting priority conservation areas for inclusion in a new or expanded protected areas network [28].

Results

Protected areas networks

We constructed 690 unique candidate protected areas satisfying our size and intactness criteria. Their mean size was 86.8 km² (SD = 4.0 km²), only very slightly larger than the target size of 85.5 km². The 500,000 candidate protected areas networks (candidate networks) included a mean of 25 (SD = 2.2) candidate protected areas added to the 62 existing protected areas. The mean candidate network area was 3138.5 km² (SD = 24.6 km²), again slightly larger than the target of 3080 km². Network ecological representativeness scores ranged from 2.76 to 7.63 (mean = 4.71, SD = 0.64) (Fig. 2). Network functional connectivity ranged from $4.21e^{+05}$ to $4.79e^{+05}$ (mean = $4.38e^{+05}$, SD = $9.42e^{+03}$) under current conditions (Fig. 2a), and from $4.41e^{+05}$ to $5.00e^{+05}$ (mean = $4.58e^{+05}$, SD = $9.51e^{+03}$) under mean future conditions (Fig. 2b). There was a negative relationship between ecological representativeness and functional connectivity, under both current (slope = -3922, $p < 0.001$, Fig. 2a) and mean future conditions (slope = -3779, $p < 0.001$, Fig. 2b).

The proposed MELCC network (Fig 1) had an ecological representativeness score of 4.65 (Fig. 2a), ~~well below~~ our sample mean. Its current and future functional connectivity were $4.59e^{+05}$ and $4.76e^{+05}$, respectively, both ~~well~~ above our sample means (Fig. 2a and 2b). However, our methodology yields many candidate networks that surpass MELCC in both representativeness and functional connectivity (Figs. 2a and 2b). We conclude that the MELCC design is not optimal with respect to these three indicators of network effectiveness.

Figure 2 approximately here

Identifying priority conservation areas

The quantile Q=0.925 (92.5 %-ile) yielded a subsample of 501 candidate protected areas scoring above the corresponding sample quantiles of all three indicators simultaneously (Figs 2a and 2b). The ecological representativeness quantile was 5.68. The quantiles for current and future functional connectivity were $4.53e^{+05}$ and $4.73e^{+05}$, respectively. All 501 selected designs had higher ecological representativeness scores than did the MELCC design. Most of these 501 designs did not exceed MELCCs functional connectivity scores, but some did (Figs. 2a and 2b; 78/501 selected designs had both current and future functional connectivity higher than did the MELCC design). That is to say, we identified some designs for the given tradeoff that outperformed MELCC’s in all three indicators.

All but one of the 690 candidate protected areas were included in at least one of the 501 selected networks. However, candidate protected area selection frequency within these networks was highly skewed (Fig 3). For example, only 32/689 candidate protected areas were included in more than 35 of the 501 selected networks (Fig 3), where 35/501 represents the inflexion point in the curve of selection frequency for the candidate protected areas (Fig. 3). These 32 frequently selected candidate protected areas were fairly widely distributed over the study region (Fig. 4), but with some evidence of spatial clustering. Notably, several of the most frequently selected are adjacent to the important caribou breeding habitats in the GNP (Fig 4).

Figure 3 and Figure 4 approximately here

Discussion

We present a methodology to define an effective protected areas network based on the trade-off between ecological representativeness and functional connectivity over time, including climate change potential impacts. Our methodology yields a proposed subset of protected areas that may be close to ideal from an environmental or conservation point of view, but which does not fully respect all constraints of the use of public lands. For example, the Gaspésie region is highly disturbed by human activities. Considering all the social and economic constraints would have reduced too much the area for potential new protected area implementations, giving little space for designing different network scenarios or exploring the limits of what is possible. However, this methodology could easily include more features in the choice of the best network scenarios, and the proposed top protected areas would then be represented as the best trade-off between all selected features. It would be possible, given data availability, to add constraints in the choice of the networks with, for example, the ecological representativeness of the future landscape under climate change, the functional connectivity of several species important for the ecosystem [59], the economic cost of excluding human activities from the proposed areas, the potential benefit with tourism if protected areas act as parks [60], or any of the constraints included by the MELCC in their scenario. Target features and constraints can be defined by local managers to help meet local biodiversity goals, and could easily be implemented in our method to improve the design of regional protected areas networks.

Network ecological representativeness

Low ecological representativeness may compromise the effectiveness or efficiency of protected areas networks. Ideally, managers would work towards achieving a better representation of the regional biodiversity when implementing new protected areas. However, to achieve biodiversity

protection, protected areas networks should aim to represent the landscape in its pre-industrial form and not to protect habitat types resulting from human disturbances. Gap analyses are a common tool to quantify ecological representativeness of extant networks and to identify under-represented features [e.g. 8,61]. Here, we adapted an alternate two-stage approach that was recently developed in Canada [48,49] to support systematic conservation planning in the boreal region. The first stage of that method used geospatial analysis tools to construct potential or candidate protected areas. Sites were then assembled into networks and a multivariate distribution matching methodology indicated networks which minimized gaps with respect to a chosen set of environmental covariates. We attempted to address some of the potential shortcomings of such a coarse filter approach by ammeding a fine filter approach that accommodates very different criteria, which is builds in an almost orthogonal dimension to the conservation problem. By doing this, we can identify tradeoffs and understand where such potential shortcomings of representativeness can be augmented to current conservation reality.

Network functional connectivity

Many studies have included connectivity in conservation planning using measures of distance or the cost of movement between protected areas [62] or applying graph and circuit theory techniques [63,64]. A novel feature of this study was that we derived network functional connectivity measures from movement simulations using an individual-based model [33,55] that reproduced known characteristics of the focal population within the study region. The model accounted for process-based complex movement behaviors (e.g., seasonal site fidelity, foray loop movement) that would be difficult to represent in static habitat-based models. Being process-based, the models reflect behavioral responses to environmental conditions and should be

preferred to make predictions under future conditions compared to models based on only observed habitat where the suppositions underlying current empirical relationships may no longer hold [65].

Accounting for climate change

It is more efficient to take future climate change impacts into account now instead of waiting for the changes to occur and then reacting [27]. To account for climate change, we defined the future functional connectivity criteria using simulated movements on hypothetical landscapes resulting from different climate change scenarios. Network effectiveness will likely depend on the realized landscape outcome which is presently unknown. Therefore, networks selected using an average functional connectivity over a wide range of climate change impacts would be sub-optimal for any particular climate change scenario. However, they might perform reasonably well under a large range of possible future environmental conditions. The differences in movement predicted for the different climate change scenarios resulted from the assumptions made about the future environmental conditions. We used this relatively simple approach as the functional connectivity differences among the scenarios were not particularly large. This suggests that using coupled climate change and vegetation change dynamic models may not dramatically improve the choice of protected areas, in this region. Furthermore, since no climate sensitive model of vegetation dynamics was available for Gaspésie natural region, using the scenario approach with averaging of results seemed to us a reasonable compromise to account for uncertainty in future conditions. Our approach could readily be adapted to cases with much greater divergence among expected future conditions, at the price of increasing the dimensionality of the tradeoff surface.

Management decisions in that case would ideally be based on weighting the relative costs of possible solutions and the probabilities associated with each alternate outcome.

Case study

The lack of functional connectivity during the last 20 years between the different major mountain summits of the Gaspésie National Park has led to a division of the caribou population into two sub-populations genetically distinct and is now jeopardizing the persistence of this isolated population [66]. Consequently, using an approach that could identify key-elements to preserve in order to maintain functional connectivity could benefit to the conservation of an endangered caribou population, especially under a changing climate.

In the Gaspésie region, there is only one caribou population occurring primarily inside the Gaspésie National Park [32,43]. In consequence, in our analyses, networks having high functional connectivity tended to include many protected areas adjacent or near to this park. On the other hand, to achieve high ecological representativeness, protected areas needed to be more or less evenly distributed over the entire region to capture habitat diversity. There was therefore a negative relationship between representation and connectivity in our case study, as others have found [e.g. 67]. It is, in general, not possible to simultaneously optimize for multiple design criteria. Our sampling-based design methodology allows to approximate a multidimensional tradeoff surface. Given the management decision about the choice of tradeoff, we can identify a large number of solutions that are close to this point. Our methodology showed how to design new protected areas for a regional network that satisfy multiple, divergent criteria to (nearly) the highest degree possible.

The MELCC network expansion proposed by the Québec government to achieve the 12% coverage target that we used as a comparison was different than the sample of candidate protected areas suggested from the selected best networks identified here (Fig[illegible]). The MELCC network achieved a lower ecological representativeness than our selected networks. However, they had to respect design criteria and constraints, like socio-economic issues or the inclusion of rare ecosystems that we did not consider. This could explain the sub-optimal ecological representativeness achieved by their network. Regarding functional connectivity, their network seems well connected from the Atlantic-Gaspésie caribou point of view. The performance of their scenario is surprisingly quite good for this feature, considering it was not explicitly part of their design. The networks we selected may not achieve such a high functional connectivity because our specific choice of intactness criteria resulted in some areas being excluded from any of our network solutions. In particular, we excluded some non-intact areas close to the Gaspésie National Park that were included in the MELCC design.

Acknowledgements

Funding for this project was provided by the Québec Fonds Verts, the Ouranos Consortium, the Centre d’étude de la forêt, Natural Sciences and Engineering Research Council Discovery Grants (to E. M. and S. C.), the Canada Research Chair’s program (to E. M.) and the French National Research Agency (ANR- 16- CE02- 0007 to S. B.). We thank the BEACONs project team (M. Houle, K. Lisgo and P. Vernier) for the provision of the custom hydrological catchments and for their help with the use of the BEACONs toolkit. We thank the Ministère de l’Environnement et de la Lutte aux Changements Climatiques du Québec (D. Boisjoly, F. Brassard and S. Benoit)

and the Ministère des Forêts, de la Faune et des Parcs du Québec for the data and documents provided.

Figure captions

Figure 1: The Gaspésie natural region with existing protected areas and the additions proposed by the government of Québec (MDDELCC 2014). Right inset: Province of Québec (Canada) with study area outlined.

Figure 2: Scatter plots of ecological representativeness against functional connectivity defined for (a) the current time period and (b) the future average conditions for the 500,000 created networks. The solid lines represent the feature values at quantile Q=0.925 (5.68 for ecological representativeness, $4.53e^{+05}$ and $4.73e^{+05}$ for current and future functional connectivity respectively). The dashed lines represent fitted linear regression models of ecological representativeness against the functional connectivity measure plotted. The white dot represent the proposed MELCC network.

Figure 3: A subsample of 501 candidate protected areas networks was selected from near one point on the tradeoff curve (Fig 2.). These candidate networks included 689 of the 690 candidate protected areas. The selection frequencies of these 689 candidate protected areas within the candidate network subsample are plotted in decreasing order. Only 32/689 candidate protected areas were included in more than 35/501 networks (threshold at the inflexion point of the curve indicated by grey line).

Figure 4: The 689 candidate protected areas included in the subsample of 501 near-Pareto-optimal networks are shown in greyscale, coloured by selection frequency, from low (light grey) to high (dark gray). The 32 candidate protected areas included in more than 35/501 networks (Fig. 3) are outlined. The existing protected areas are shown in black.

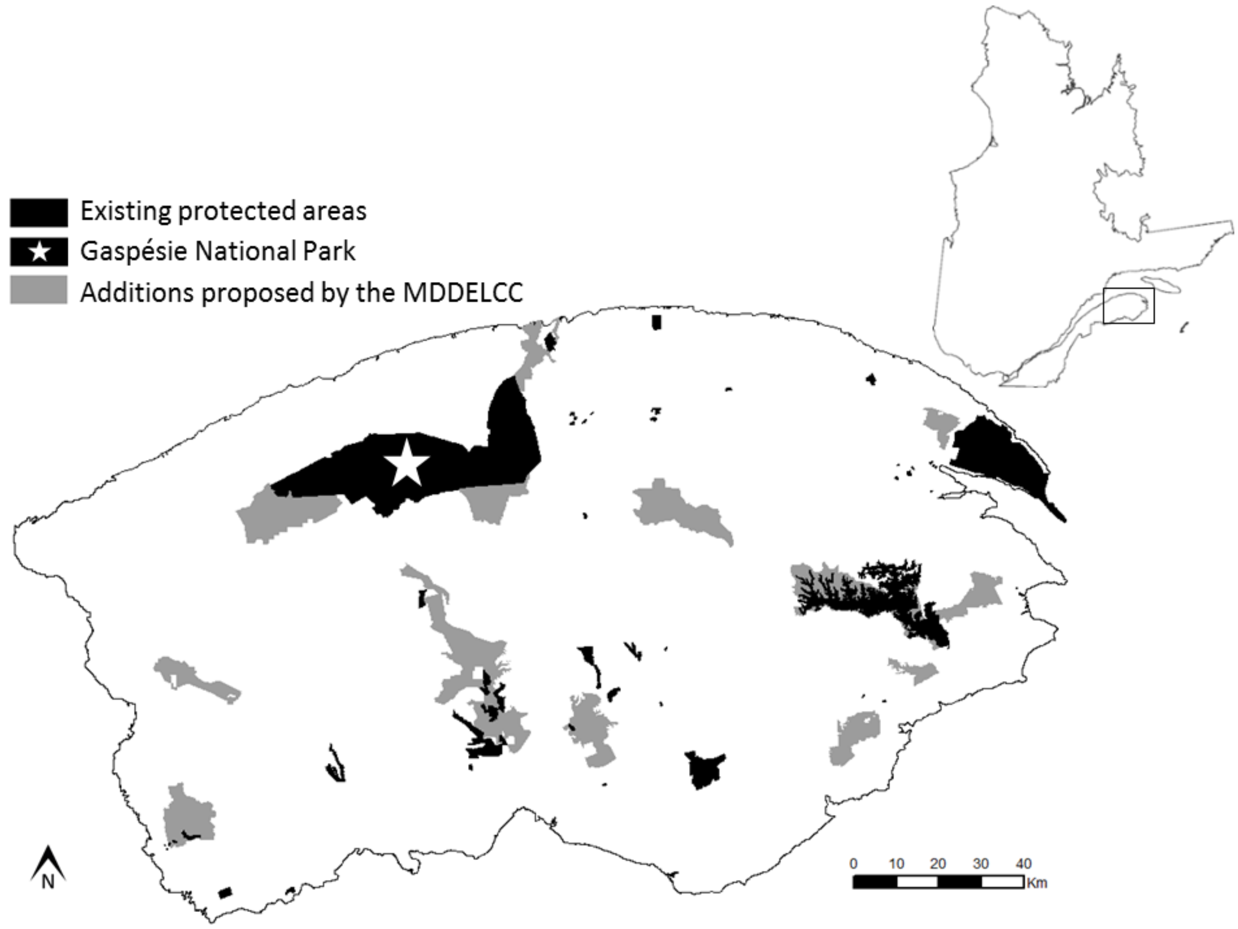
Existing protected areas
Gaspésie National Park
Additions proposed by the MDDELCC
N
0 10 20 30 40
Km

Figure 1

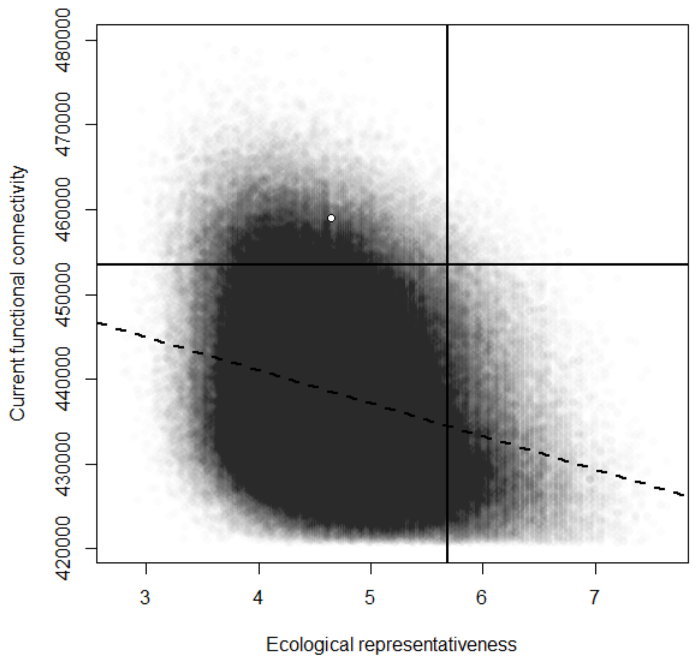
Current functional connectivity
480000
470000
460000
450000
440000
430000
420000
3
4
5
6
7
Ecological representativeness

a)

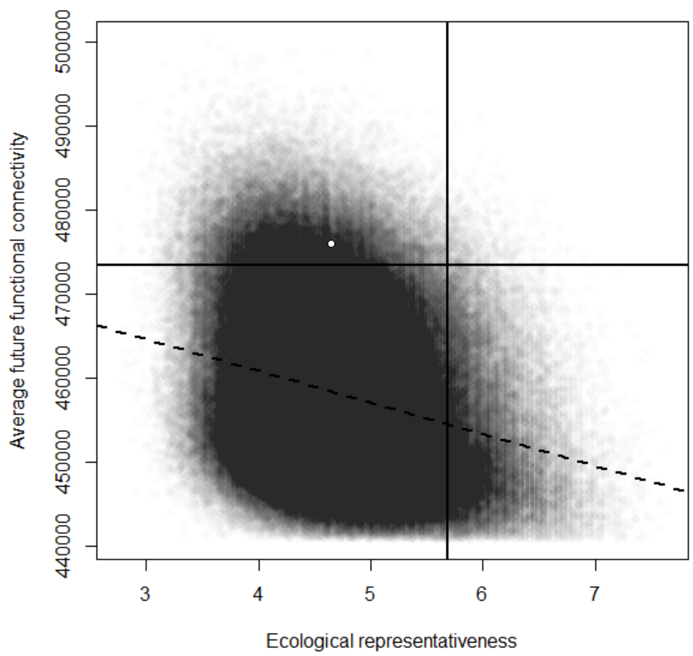
Average future functional connectivity
500000
490000
480000
470000
460000
450000
440000
3
4
5
6
7
Ecological representativeness

b)

Figure 2

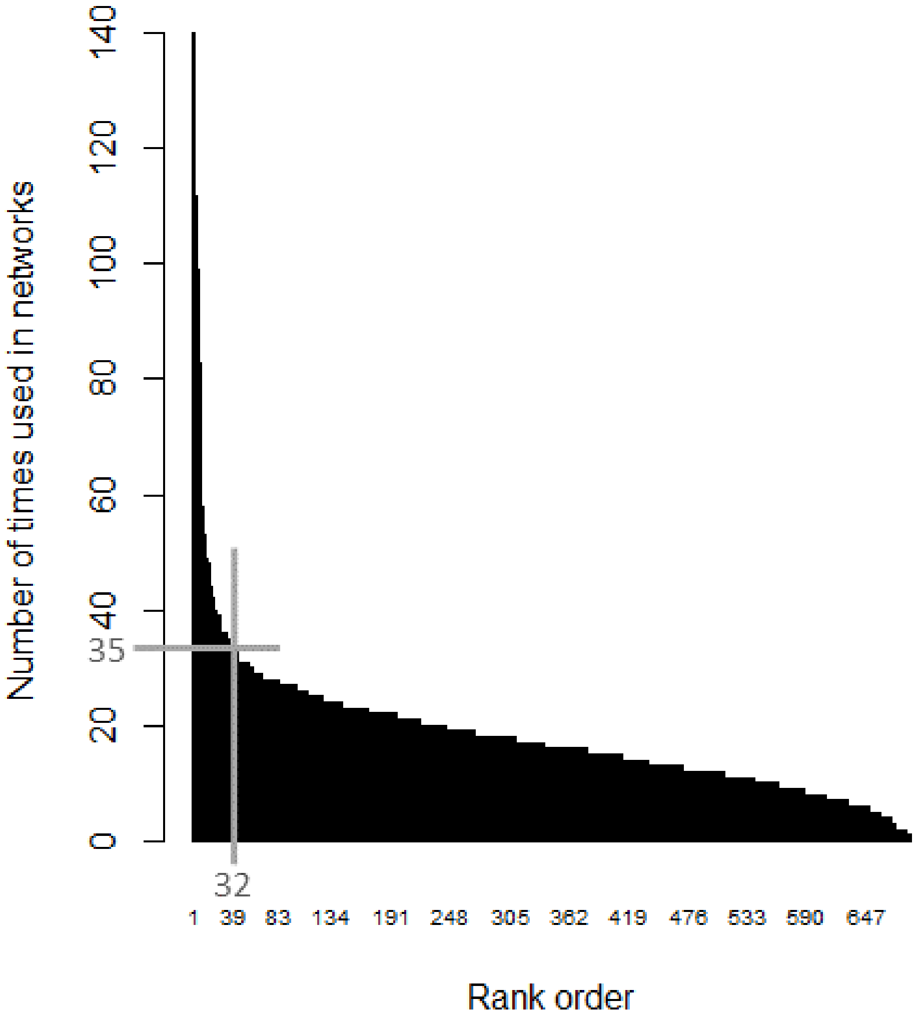
Number of times used in networks
0
20
35
40
60
80
100
120
140
32
1 39 83 134 191 248 305 362 419 476 533 590 647
Rank order

Figure 3

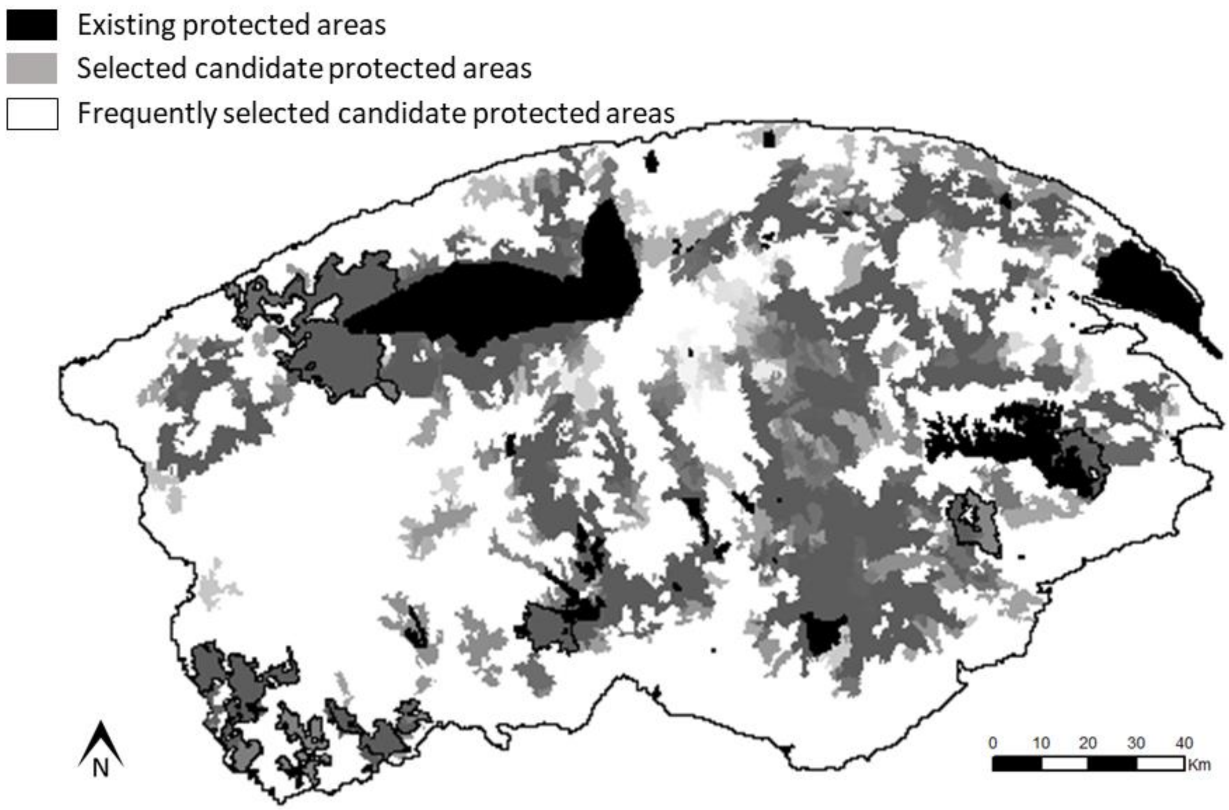
Existing protected areas
Selected candidate protected areas
Frequently selected candidate protected areas
N
0 10 20 30 40
Km

Figure 4

References

1. Secretariat of the Convention on Biological Diversity. Global biodiversity outlook 2. Montreal, Canada : Secretariat of the Convention on Biological Diversity; 2006.

2. Tylianakis JM, Laliberté E, Nielsen A, Bascompte J. Conservation of species interaction networks. Biol Conserv. 2010;143(10):2270–2279.

3. Spooner FE, Pearson RG, Freeman R. Rapid warming is associated with population decline among terrestrial birds and mammals globally. Glob Chang Biol. 2018;24(10):4521–4531.

4. Coetzee BW, Gaston KJ, Chown SL. Local scale comparisons of biodiversity as a test for global protected area ecological performance: a meta-analysis. PloS one. 2014;9(8):e105824.

5. Ervin J. Protected area assessments in perspective. Bioscience. 2003;53(9):819–822.

6. Geldmann J, Barnes M, Coad L, Craigie ID, Hockings M, Burgess ND. Effectiveness of terrestrial protected areas in reducing habitat loss and population declines. Biol Conserv. 2013;161:230–238.

7. Gaston KJ, Jackson SF, Cantú-Salazar L, Cruz-Piñón G. The ecological performance of protected areas. Annu Rev Ecol Evol Syst. 2008;39:93–113.

8. Rodrigues AS, Andelman SJ, Bakarr MI, Boitani L, Brooks TM, Cowling RM, et al. Effectiveness of the global protected area network in representing species diversity. Nature. 2004;428(6983):640.

9. Stewart FE, Darlington S, Volpe JP, McAdie M, Fisher JT. Corridors best facilitate functional connectivity across a protected area network. Sci Rep. 2019;9(1):1–9.

10. Nuñez TA, Lawler JJ, McRae BH, Pierce DJ, Krosby MB, Kavanagh DM, et al. Connectivity planning to address climate change. Conserv Biol. 2013;27(2):407–416.

11. Beaver D, Llewellyn G. Designing a comprehensive, adequate and representative (CAR) network of marine protected areas for Australia’s Commonwealth waters. Ultimo, Australia: WWF; 2009 Feb.

12. Kukkala AS, Moilanen A. Core concepts of spatial prioritisation in systematic conservation planning. Biol Rev Camb Philos Soc. 2013;88(2):443–464.

13. Scott JM, Davis FW, McGhie RG, Wright RG, Groves C, Estes J. Nature reserves: Do they capture the full range of America’s biological diversity? Ecol Appl. 2001;11(4):999–1007.

14. Joppa LN, Pfaff A. High and far: biases in the location of protected areas. PloS one. 2009;4(12):e8273.

15. Asaad I, Lundquist CJ, Erdmann MV, Costello MJ. Ecological criteria to identify areas for biodiversity conservation. Biol Conserv. 2017;213:309–316.

16. Taylor PD, Fahrig L, Henein K, Merriam G. Connectivity is a vital element of landscape structure. Oikos. 1993;571–573.

17. Baguette M, Van Dyck H. Landscape connectivity and animal behavior: functional grain as a key determinant for dispersal. Landsc Ecol. 2007;22(8):1117–1129.

18. Kadoya T. Assessing functional connectivity using empirical data. Popul Ecol. 2009;51(1):5–15.

19. Andrello M, Jacobi MN, Manel S, Thuiller W, Mouillot D. Extending networks of protected areas to optimize connectivity and population growth rate. Ecography. 2015;38(3):273–282.

20. Urban MC. Accelerating extinction risk from climate change. Science. 2015;348(6234):571–573.

21. Jantz SM, Barker B, Brooks TM, Chini LP, Huang Q, Moore RM, et al. Future habitat loss and extinctions driven by land-use change in biodiversity hotspots under four scenarios of climate-change mitigation. Conserv Biol. 2015;29(4):1122–1131.

22. Loarie SR, Duffy PB, Hamilton H, Asner GP, Field CB, Ackerly DD. The velocity of climate change. Nature. 2009;462(7276):1052.

23. Dawson TP, Jackson ST, House JI, Prentice IC, Mace GM. Beyond predictions: biodiversity conservation in a changing climate. Science. 2011;332(6025):53–58.

24. Chen I-C, Hill JK, Ohlemüller R, Roy DB, Thomas CD. Rapid range shifts of species associated with high levels of climate warming. Science. 2011;333(6045):1024–1026.

25. Lawler JJ, Ruesch AS, Olden JD, McRae BH. Projected climate-driven faunal movement routes. Ecol Lett. 2013;16(8):1014–1022.

26. Vos CC, Berry P, Opdam P, Baveco H, Nijhof B, O'Hanley J, et al. Adapting landscapes to climate change: examples of climate-proof ecosystem networks and priority adaptation zones. J Appl Ecol. 2008;45(6):1722–1731.

27. Hannah L, Midgley G, Andelman S, Araújo M, Hughes G, Martinez-Meyer E, et al. Protected area needs in a changing climate. Front Ecol Environ. 2007;5(3):131–138.

28. Klein C, Wilson K, Watts M, Stein J, Berry S, Carwardine J, et al. Incorporating ecological and evolutionary processes into continental-scale conservation planning. Ecol Appl. 2009;19(1):206–217.

29. Calkin DE, Montgomery CA, Schumaker NH, Polasky S, Arthur JL, Nalle DJ. Developing a production possibility set of wildlife species persistence and timber harvest value. Can J For Res. 2002;32(8):1329–1342.

30. Arthur JL, Camm JD, Haight RG, Montgomery CA, Polasky S. Weighing conservation objectives: maximum expected coverage versus endangered species protection. Ecol Appl. 2004;14(6):1936–1945.

31. COSEWIC. Designatable units for caribou (Rangifer tarandus) in Canada. Ottawa, Canada: Committee on the Status of Endangered Wildlife in Canada; 2011.

32. Morin M. Inventaire aérien de la population de caribous de la Gaspésie (Rangifer tarandus caribou) – Automne 2018. Ministère des Forêts, de la Faune et des Parcs; 2018.

33. Bauduin S, McIntire E, St-Laurent M-H, Cumming S. Overcoming challenges of sparse telemetry data to estimate caribou movement. Ecol Modell. 2016;335:24–34.

34. Bouchard M-A. Portrait du réseau d'aires protégées au Québec - Analyse de carence écorégionale, Gaspésie (Terrestre). Ministère du Développement Durable de l'Environnement et de la Lutte aux Changements Climatiques;2014.

35. Saucier J-P, Grondin P, Robitaille A, Bergeron J-F. Zones de végétation et les domaines bioclimatiques du Québec. Ministère des ressources naturelles,de la Faune et des Parcs; 2003.

36. Brassard F, Bouchard AR, Boisjoly D, Poisson F, Bazoge A, Bouchar, M-A, et al. Portrait du réseau d'aires protégées au Québec: période 2002-2009. Ministère du Développement durable, de l'Environnement et des Parcs du Québec; 2010.

37. LeSage L, Paquin P. Historique, géographie physique et biogéographie du Parc de conservation de la Gaspésie, Québec. Proc ent Soc Ont. 2000;131:17–66.

38. Species at Risk Public Registry [Internet]. Environment Canada; c2019 [cited 2019 06 18]. Available from: http://www.sararegistry.gc.ca/.

39. Lesmerises F, Déry F, Johnson CJ, St-Laurent M-H. Spatiotemporal response of mountain caribou to the intensity of backcountry skiing. Biol Conserv. 2018;217:149–156.

40. Boudreau M. Impacts de 25 ans d'aménagement forestier intensif sur l'habitat du caribou de la Gaspésie et de ses prédateurs. M.Sc. Thesis, Université du Québec à Rimouski. 2017. Available from: http://semaphore.uqar.ca/1289/.

41. Frenette J. Démographie et viabilité de la population de caribous de la Gaspésie-Atlantique. M.Sc. Thesis, Université du Québec à Rimouski. 2017. Available from: http://semaphore.uqar.ca/1293/.

42. Ouellet J-P, Ferron J, Sirois L. Space and habitat use by the threatened Gaspé caribou in southeastern Quebec. Can J Zool. 1996;74(10):1922–1933.

43. Mosnier A, Ouellet J-P, Sirois L, Fournier N. Habitat selection and home-range dynamics of the Gaspé caribou: a hierarchical analysis
Can J Zool. 2003;81(7):1174–1184.

44. Mosnier A, Boisjoly D, Courtois R, Ouellet J-P. Extensive predator space use can limit the efficacy of a control program. J Wildl Manage. 2008;72(2):483–491.

45. Dumais C, Ropars P, Denis M-P, Dufour-Tremblay G, Boudreau S. Are low altitude alpine tundra ecosystems under threat? A case study from the Parc National de la Gaspésie, Québec. Environmental Research Letters. 2014;9(9):094001.

46. Renard S, Isabel C, McIntire E. Le vent, un facteur déterminant pour le maintien de la toundra alpine. In : Bulletin de conservation 2014-2015. Québec, Canada : Société des Établissements de Plein Air du Québec; 2015. p. 27-30

47. Périé C, de Blois S, Lambert M-C, Casajus N. Effets anticipés des changements climatiques sur l'habitat des espèces arborescentes au Québec. Ministère des Ressources naturelles du Québec; 2014.

48. Saucier F. Développement d'une approche de planification systématique pour l'élaboration de réseaux de conservation représentatifs faits d'aires protégées qui visent la persistance de la biodiversité à long terme : étude de cas au Moyen Nord du Québec. M.Sc. Thesis, Université Laval. 2011. Available from: https://corpus.ulaval.ca/jspui/handle/20.500.11794/22949.

49. BEACONs. The Canadian BEACONs Project [Internet]. The BEACONs' team; [cited 2018 07 18]. Available from: http://beaconsproject.ca/.

50. Linke S, Norris RH, Pressey RL. Irreplaceability of river networks: towards catchment-based conservation planning. J Appl Ecol. 2008;45(5):1486–1495.

51. Hermoso V, Filipe AF, Segurado P, Beja P. Effectiveness of a large reserve network in protecting freshwater biodiversity: a test for the Iberian Peninsula.
Freshw Biol. 2015;60(4):698–710.

52. Adams VM, Setterfield SA, Douglas MM, Kennard MJ, Ferdinands K. Measuring benefits of protected area management: trends across realms and research gaps for freshwater systems. Philos Trans R Soc Lond **B** Biol Sci. 2015;370(1681):20140274.

53. Lowe WH, Likens GE. Moving headwater streams to the head of the class. BioScience. 2005;55(3):196–197

54. Polak T, Watson JE, Bennett JR, Possingham HP, Fuller RA, Carwardine J. Balancing ecosystem and threatened species representation in protected areas and implications for nations achieving global conservation goals. Conserv Lett. 2016;9(6):438–445.

55. Bauduin S, McIntire E, St-Laurent M-H, Cumming SG. Compensatory conservation measures for an endangered caribou population under climate change. Sci Rep. 2018;8(1):16438.

56. Logan T. Scénarios climatiques pour les régions naturelles de la péninsule de la Gaspésie et la dépression de La Tuque. Consortium Ouranos; 2012.

57. Gray DR. The relationship between climate and outbreak characteristics of the spruce budworm in eastern Canada. Clim Change. 2008;87(3–4):361–383.

58. Régnière J, St-Amant R, Duval P. Predicting insect distributions under climate change from physiological responses: spruce budworm as an example. Biol Invasions. 2012;14(8):1571–1586.

59. Minor ES, Urban DL. A graph-theory framework for evaluating landscape connectivity and conservation planning. Conserv Biol. 2008;22(2):297–307.

60. Mayer M. Can nature-based tourism benefits compensate for the costs of national parks? A study of the Bavarian Forest National Park, Germany. Journal of Sustainable Tourism. 2014;22(4):561–583.

61. Wiersma YF, Nudds TD. Efficiency and effectiveness in representative reserve design in Canada: the contribution of existing protected areas. Biol Conserv. 2009;142(8):1639–1646.

62. Sundblad G, Bergström U, Sandström A. Ecological coherence of marine protected area networks: a spatial assessment using species distribution models. J Appl Ecol. 2011;48(1):112–120.

63. Baldwin RF, Perkl RM, Trombulak SC, Burwell WB. Modeling ecoregional connectivity. In: Trombulak SC, Baldwin RF, editors. Landscape-scale conservation planning. Dordrecht, Netherlands: Springer; 2010. p. 349–367.

64. Rayfield B, Fortin M-J, Fall A. Connectivity for conservation: a framework to classify network measures. Ecology. 2011;92(4):847–858.

65. Bélisle M. Measuring landscape connectivity: the challenge of behavioral landscape ecology. Ecology. 2005;86(8):1988–1995.

66. Pelletier F, Turgeon G, Bourret A, Garant D, St-Laurent M-H. Genetic structure and effective size of an endangered population of woodland caribou. Conserv Genet. 2019;20(2):203–213.

67. Almany GR, Connolly SR, Heath DD, Hogan JD, Jones GP, McCook LJ, et al. Connectivity, biodiversity conservation and the design of marine reserve networks for coral reefs. Coral Reefs. 2009;28(2):339–351.

Figure 1

Click here to download Figure Bauduin et al_PLoS One_Fig1.tif

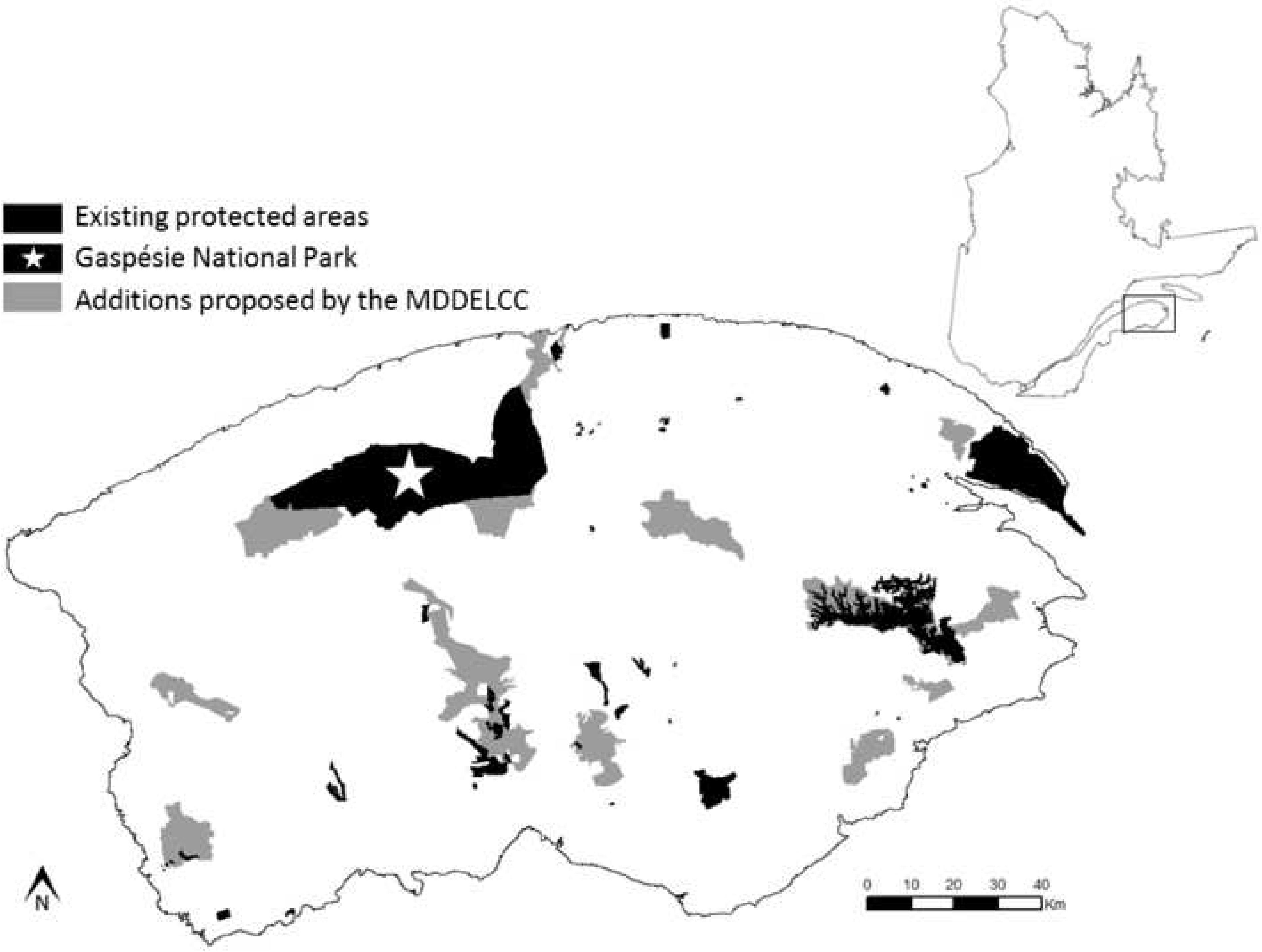

Figure 2a

Click here to download Figure Bauduin et al_PLoS One_Fig2a.tif

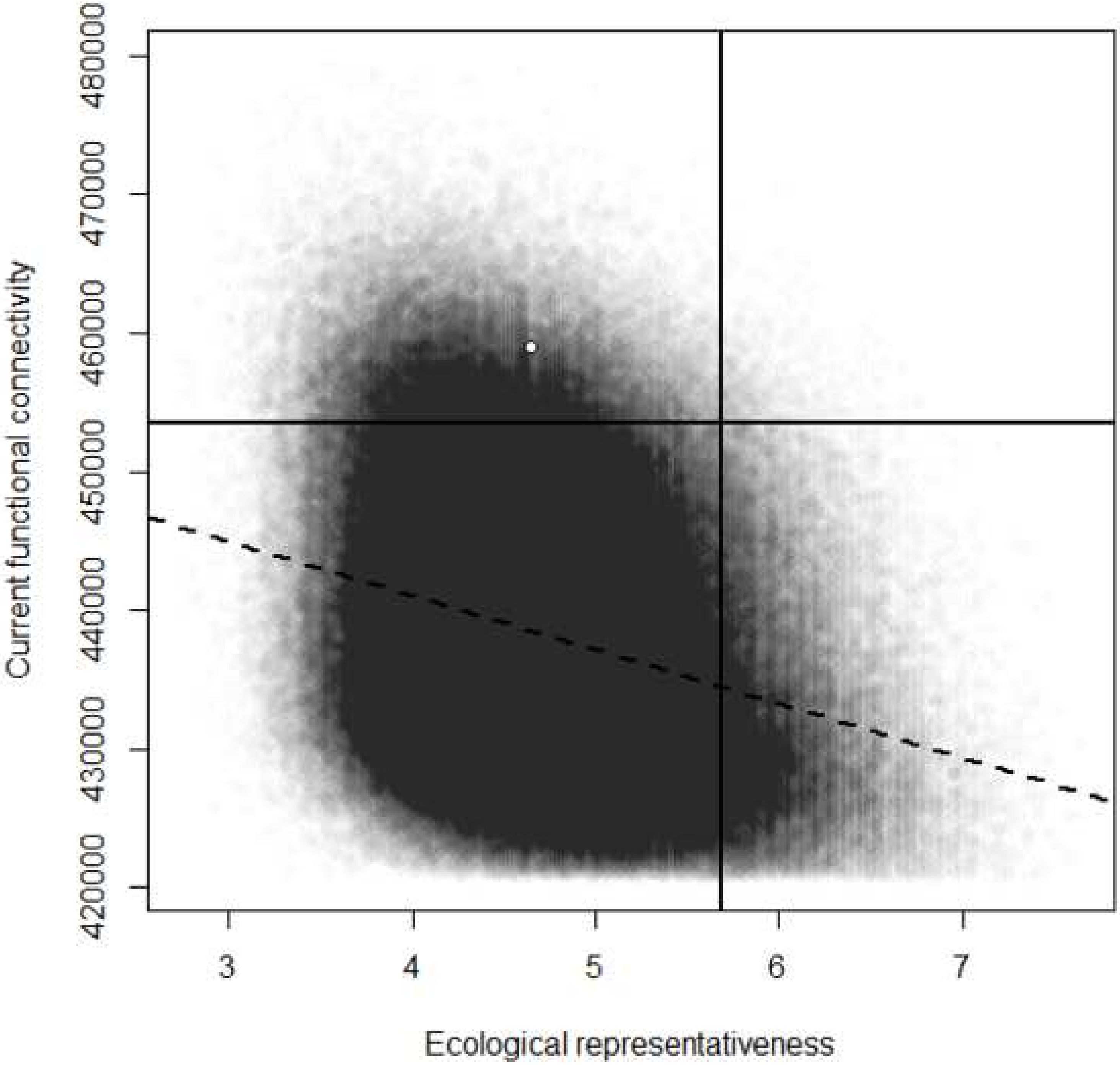

Figure 2b

Click here to download Figure Bauduin et al_PLoS One_Fig2b.tif

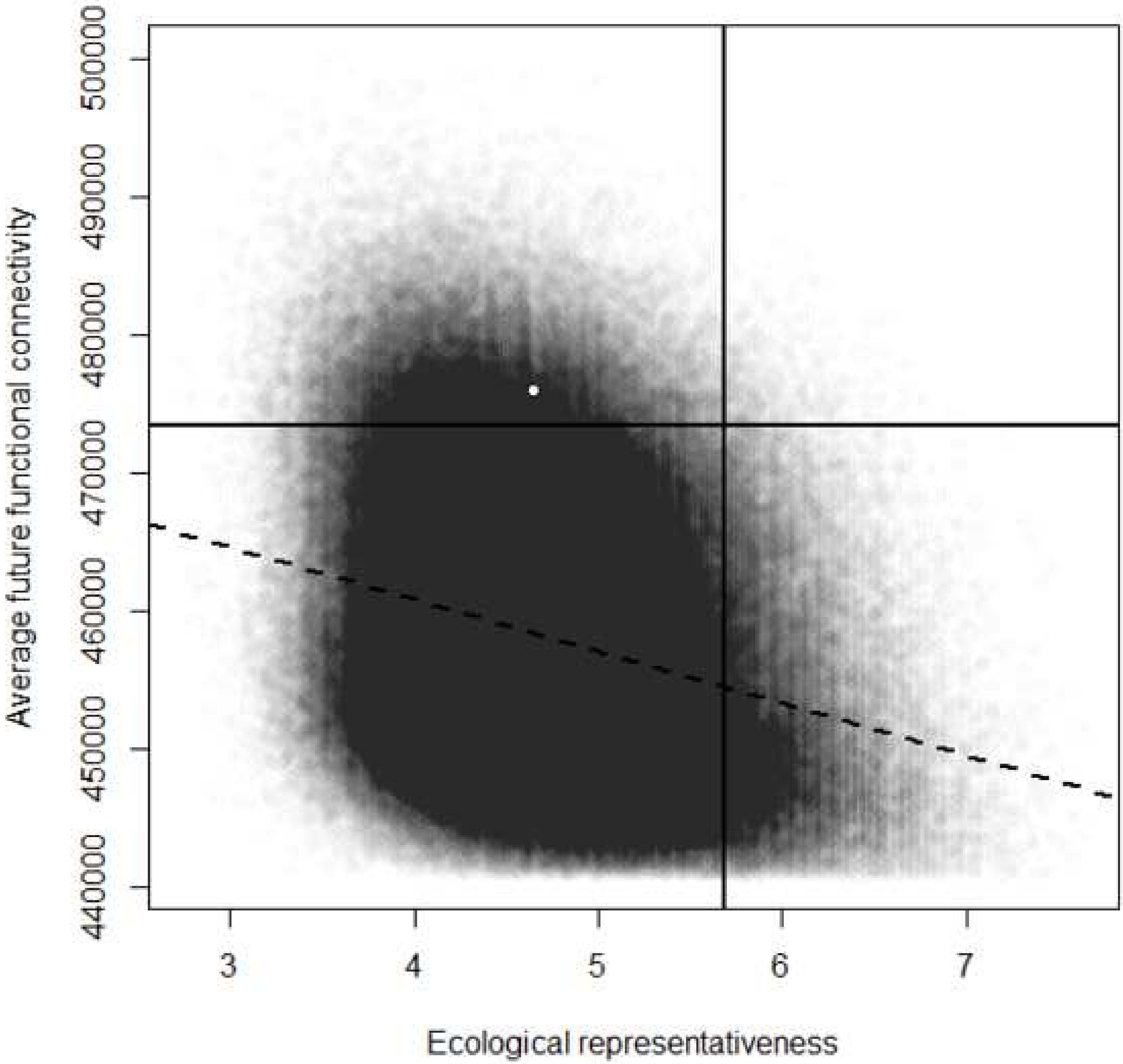

Figure 3

Click here to download Figure Bauduin et al_PLoS One_Fig3.tif

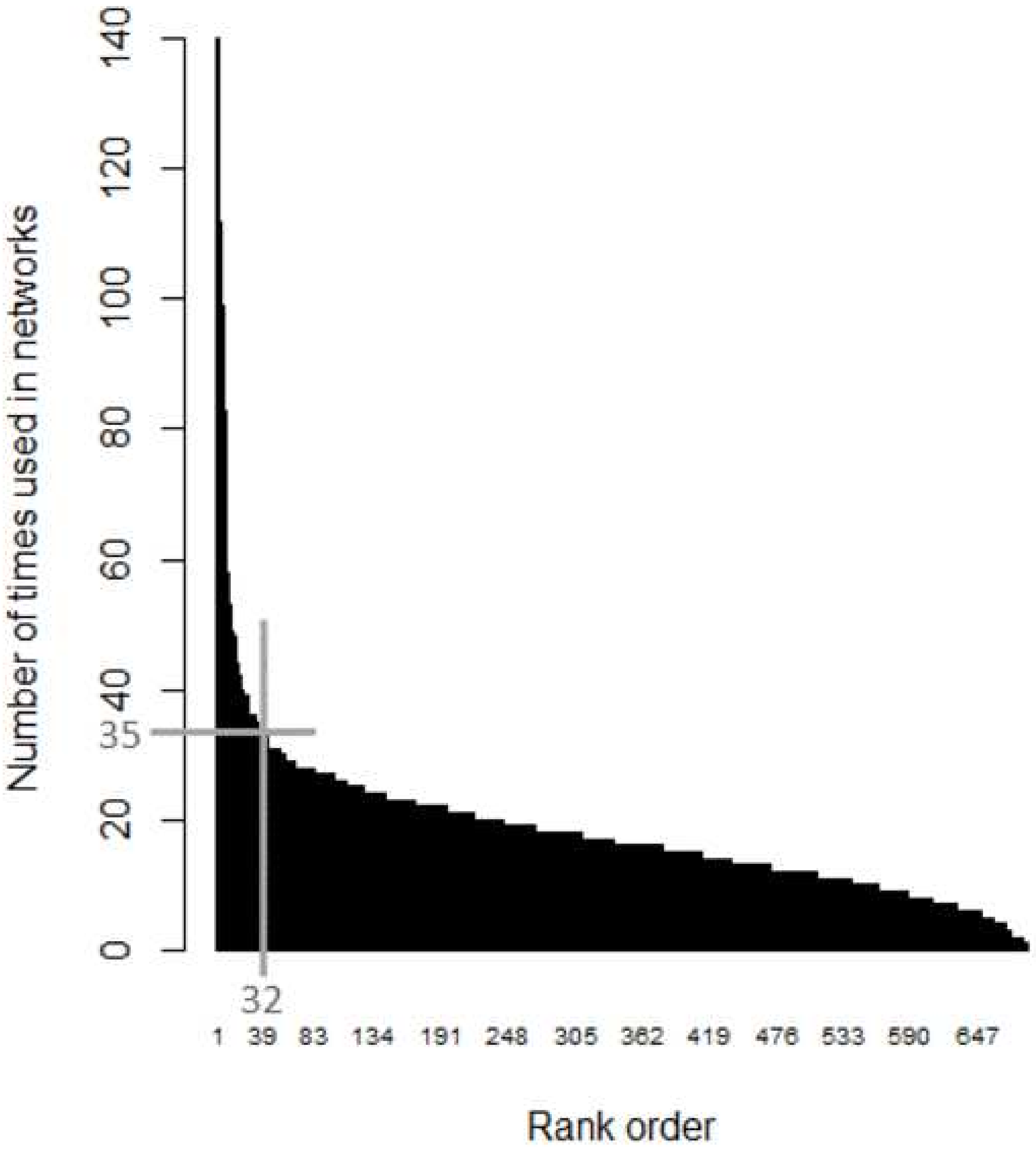

Figure 4

Click here to download Figure Bauduin et al_PLoS One_Fig4.tif

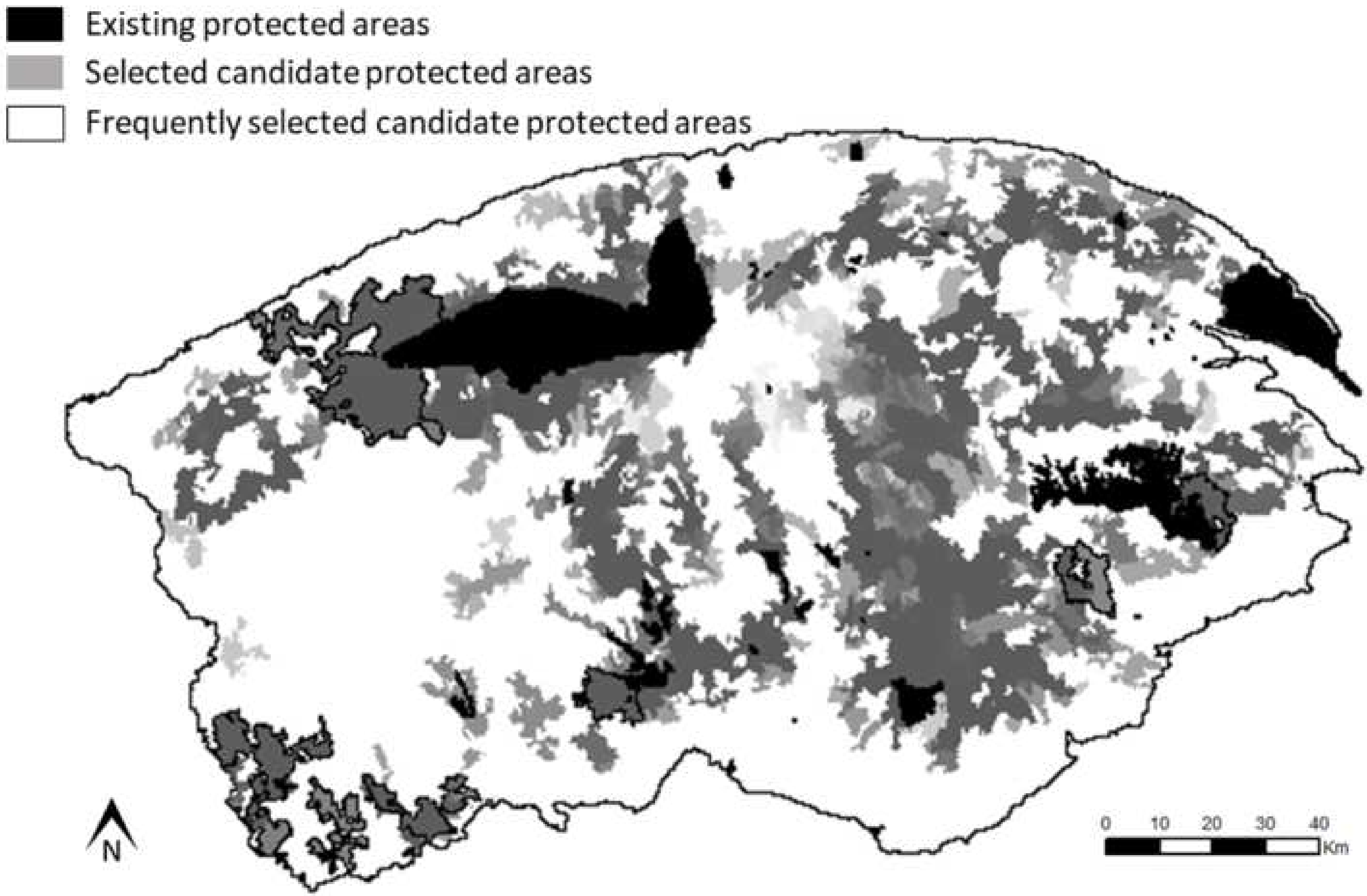

Click here to access/download
Supporting Information
Bauduin et al_PLoS One_SuppInfo_SI1.docx
