# Supplementary material for: Integrating functional connectivity in designing networks of protected areas under climate change: A caribou case-study
Source: PLoS One. 2020 Sep 30;15(9):e0238821. doi: 10.1371/journal.pone.0238821 (PMC7526922; doi:10.1371/journal.pone.0238821)
Supplement: Attachment — Submitted filename: Responses to Reviewers_PONE-D-20-04608_R1.pdf [file pone.0238821.s007.pdf]

Integrating functional connectivity in designing networks of protected areas under climate change: a caribou case-study

(MS # PONE-D-20-04608.R2)

Responses to reviewers' comments

Please find below our responses to the reviewer's comments; the line numbers in our responses refer to the revised version (clean copy) of our manuscript.

Please note that we have also uploaded a version of our revised manuscript with all modifications highlighted in "Track Change".

REVIEWER #2

COMMENT #1. Thank you for your efforts to revise the manuscript in response to my previous review. The result is a clearer work that communicates the important approach you have taken. There remain, however, a few issues that should be addressed prior to acceptance of the manuscript. I hope that these will be easy to update and that the manuscript can then be accepted.

> ***OUR RESPONSE:*** Thanks for the time invested in reviewing our manuscript and helping us, your comments were greatly appreciated.

COMMENT #2. L.197-198. Both reviewers questioned giving an equal weight to each disturbance type, which suggests other readers will as well. Having read the authors' responses to our comments, I now understand the authors' reasoning behind this decision and suggest that a few sentences be added to clarify this for other readers. For example (adapting from the response to Comment 14): "…This gives equal weight to each disturbance type. While this is likely unrealistic for any given species, catchment intactness was intended to be relevant to biodiversity more broadly. Equal weighting reflects differing

responses across species. For example, agricultural fields have a large impact on caribou but less impact on meso-carnivores (Toews et al. 2018). Even urban areas can be less avoided by some species (e.g., birds, insects) relative to either heavily forested areas or highways and their surroundings. Equal weighting thus represents a conservative assumption."

OUR RESPONSE: We have added some text to this section, adapted from your suggestions (lines 206-211).

COMMENT #3. I greatly appreciate the authors adding Fig 2 and making modifications to Fig 5 and the Methods description in response to my comments. This greatly helped in clarifying the approach used in their model. I still am a little confused, however, by what size threshold was used in creating candidate protected areas. In combination with Fig 2, L.209-213 indicate that starting with the seed catchments – which should be intact headwater catchments (presumably only on public land, see next point) – additional catchments along the stream network that met the intactness criteria of > median intactness were added sequentially until the target size of 85.5 sq km was met or slightly exceeded. At that point the set of combined catchments was considered a candidate protected area. The sentence from L.213-215 then seems redundant, simply restating what was just described so I suggest removing it. But then L.216-218 indicate that candidate protected areas with a total area above 31.2 sq km were selected. This is where I am confused. If the target size was 85.5 sq km and catchments were added until this was met or exceeded, what was the 31.2 sq km size used for? Should not all candidate protected areas have exceeded this size? I do not see this clearly explained. This needs to be clarified as the difference between these two target sizes affects the ability of readers to understand the criteria used and to replicate the study.

OUR RESPONSE: We have revised the text to clarify and to motivate the distinction between the target and minimum sizes (Lines 218-227).

COMMENT #4. L.209. Fig 2 says intact headwater catchments on public land were used as seeds, but this line does not specify that the seeds had to be on public land. If this was a constraint it should be indicated here as well.

OUR RESPONSE: We specify in the preceding paragraph that "intact catchments" were defined so as to exclude catchments on private lands. We have modified Fig. 2 to remove the reference to this case-study-specific detail, so that the caption is now consistent with the text at the former L. 209. This avoids repeating the same detail in three places.

COMMENT #5. L.316,321. These references to Fig 2 seem a little strange to me as what is being described is not clearly pictured in Fig 2. There is some text that indicates seeking tradeoffs, but mostly I found myself looking for explanations of what was meant by "a subsample of 0.1% of the candidate networks that were highly ranked under all three criteria" and "using as the frequency threshold the inflection point in the proportional frequency curve," neither of which seem to be indicated in Fig 2. I suggest removing these references to the figure as they really are not necessary here.

> ***OUR RESPONSE:*** We agree and have removed the two references to Figure 2 (lines 325 and 327). This information actually refers more to Figure 3 but as Figure 3 presents results, we won't refer to it in the Methods. The subsection in question (Lines 324-332) has been revised to improve clarity.

COMMENT #6. L.354. A reference is given to S3 Supporting Information to support the statement that "Most of these 501 designs did not exceed MELCCs functional connectivity scores, but some did." However, MELCC is never mentioned in Supplementary Information S3, nor are specific values of the displayed protected areas or even a labelled color ramp of values given that would allow the reader to evaluate the claim made in the paper, so this reference seems inappropriate. Instead, S3 reports statistics based on a single optimality criterion only, which is not discussed anywhere in the main text. While I suggested making figures like this in my previous review, they need to be at least briefly introduced in the text and then given further description in the supplement, or else removed entirely.

> ***OUR RESPONSE:*** The material in S3 Supporting Information is indeed unrelated to the statement in question (lines 353-356 in the previous version). We now refer instead to Figure 3, which does support the statement (lines 368-370 of the revised version), and made also some revisions to the paragraph to improve notation and clarity (lines 365-384). With regards to S3, it is now motivated in the Methods (Lines 334-338), and references in the Results (Lines 379-384) and Discussion (Lines 470-472); the text of S3 itself has also been expanded, as requested

COMMENT #7. L.396-399. I am unclear what is meant by "We attempted to address some of the potential shortcomings of such a coarse filter approach by amending a fine filter approach that accommodates very different criteria, which is builds in an almost orthogonal dimension to the conservation problem." Which fine filter approach is meant? I first thought it was a reference to the caribou IBM, but considering that this is all under a heading of "Network ecological representativeness" and the caribou model fits under the next section, "Network functional connectivity," I am left unclear by what the authors

consider the fine filter approach? This is the only place in the manuscript where this term shows up other than the key words. What is meant by the fine filter and coarse filter approach need to be clearly described. Likewise, the potential shortcomings of the coarse filter approach should be described, at least in brief. Then it should be explained how the fine filter approach accommodates a different criterion and builds in an orthogonal dimension to the problem. As it stands, none of this is clear to me.

> ***OUR RESPONSE:*** The references to fine and course filter approaches were superfluous and have been deleted.

COMMENT #8. L.486. MDDELCC should be defined in the figure caption so that the figure can stand on its own. This is especially important because while the acronym MELCC is regularly used in the paper, MDDELCC does not show up anywhere in the main text or supplements, nor is there an MDDELCC 2014 reference in the References. The citation for this needs to be clarified.

> ***OUR RESPONSE:*** Our use of "MDDELCC" as a citation key to Bouchard (2014) was incorrect. In the revised caption of Figure 1, we now use the journal's numerical citation format. The acronym MDDELCC no longer appears in the caption or the Figure's legend. We note that the responsible ministry has changed names several times since this project was initiated. We now refer throughout to "the Ministry", as per the revised Introduction (Lines 161-162).

COMMENT #9. L.505. Use of color in this figure improves clarity over the initial version. However, the greenscale color ramp used to show selection frequency of candidate protected areas in Figure 5 is subtle enough that it remains difficult to discern differences among many of the protected areas. It would be helpful to use a different color ramp with greater contrast. This also applies to the figures in Supplementary Information S3.

> ***OUR RESPONSE:*** We changed the color ramp for Figure 5 (Lines 529-532) and for the Figures in S3 Supporting Information.

COMMENT #10. Finally, a number of typos or grammatical issues remain in the manuscript that should be addressed prior to publication. These include:

- L.21. Change "disturbanes" to "disturbances"

  OUR RESPONSE: Corrected.

- L.133. Change “potental” to “potential”

  OUR RESPONSE: Corrected.

- L. 135. Change “and well as” to “as well as a”

  OUR RESPONSE: Corrected.

- L. 135. Remove extra space between “while” and “also”

  OUR RESPONSE: Corrected.

- L. 140. I suggest changing “has been” to “was”

  OUR RESPONSE: Done.

- L.167. Change “it” to “this caribou population” to clarify for those reading quickly that “it” does not refer to the park, as the subject of the previous sentence was Gaspésie National Park.

  OUR RESPONSE: Done.

- L.177. Change “completing” to “complementing”

  OUR RESPONSE: Done.

- L.271. Remove the comma from “model, process”

  OUR RESPONSE: Corrected.

- L.289. Remove the second “with” from “combined with with predicted”

  OUR RESPONSE: Corrected.

- L.438-439. Rephrase to “two genetically distinct sub-populations”

  OUR RESPONSE: Done.

- L.489. This should be changed to either “assembling a candidate protected areas network” or to “assembling candidate protected areas networks”. It was not clear to me which the authors intend.

  OUR RESPONSE: This comment refers to the caption of Figure 2. Our intended meaning was the second you mentioned “assembling candidate protected areas networks”. The caption has been revised (Lines 512-515).

- L.491. Change “middel" to “middle”

  OUR RESPONSE: Corrected.

- L.491. Change “hydroligically” to “hydrologically”

  OUR RESPONSE: Corrected.

- L.496. Insert a comma after “connectivity”

  OUR RESPONSE: Corrected.

REVIEWER #3

COMMENT #11 – Title. The research reported in this manuscript seems highly relevant to conservation planning for the Atlantic-Gaspésie caribou population. However, I think the new title of the manuscript should be changed a little bit. Hence, I suggest revising the title. A suggested title is “Integrating functional connectivity in designing networks of protected areas under climate change: a caribou case-study”.

OUR RESPONSE: We have revised the title much as you suggested.

COMMENT #12. L.348 I suggest you change the name “Identifying priority conservation areas” from the results to “Priority conservation areas” as it is the same as the title from the methods (L.310) and can be confusing.

OUR RESPONSE: We have modified the section heading according to your suggestion (line 363).

COMMENT #13. L.504 “inflection” instead of “inflexion”.

OUR RESPONSE: Done (line 527)
