# Supplementary material for: Integrating functional connectivity in designing networks of protected areas under climate change: A caribou case-study
Source: PLoS One. 2020 Sep 30;15(9):e0238821. doi: 10.1371/journal.pone.0238821 (PMC7526922; doi:10.1371/journal.pone.0238821)
Supplement: Attachment — Submitted filename: Review of PONE-D-20-04608.docx [file pone.0238821.s005.docx]

Review of PONE-D-20-04608

This manuscript proposes an approach to identifying protected area networks using simulation modeling that seek to balance multiple ecological criteria. The authors focus on ecological representativeness and functional connectivity as their metrics for optimization, with the former relating to broad landscape features and the latter focusing on an endangered species in Canada – the Atlantic-Gaspésie caribou population. They consider potential networks of protected areas under both current conditions and future conditions of climate change and timber management. The paper appears methodologically sound, though several of the descriptions should be clarified and supported with figures to make the approach clearer to readers. With the addressing of the issues below, I think this paper would make a worthy contribution to PLoS ONE.

The primary improvement needed for this paper is clarification of the methodology and support with additional figures. This paper really is focused around the details of its methods as it proposes a detailed methodology for conducting optimization. However, there are multiple places where methods are difficult to determine based on the information as currently organized, especially for those unfamiliar with the BEACONs approach. Given the spatial nature of the proposed simulation approach it is especially difficult to visualize the steps of the method from the description given. I had to read the methods multiple times to wrap my head around the steps the authors take to simulate candidate protected area networks. As best I can tell, the procedure that was used was to:

1. Identify a set of initial “seed” catchments
   a. Defined here as all intact headwater catchments
2. For each seed catchment, add contiguous catchments that are hydrologically connected and meet minimum size and intactness criteria
   a. Stop adding catchments when target size is achieved or no more catchments that meet the criteria are available to be added
   b. Define the set of catchments associated with a given seed as a “candidate protected area” if its total area is greater than 31.2 sq km.
3. From the full set of candidate protected areas, generate “candidate protected area networks” by randomly selecting candidate protected areas and adding them to the existing protected area network until the total area exceeds 3080 sq km.
   a. Repeat this step 500,000 times to generate many possible candidate protected area networks.

The paper would greatly benefit from clearly stating the above steps and from addition of one or two figures clarifying these steps. How protected area networks are generated is central to the methods and validity of the paper. I recommend the steps above be clearly indicated in the text and perhaps in a flowchart, with one or two figures displaying the corresponding spatial depictions:

- All seed catchments across the study area, shown in relation to existing protected areas.
- One candidate protected area, with each of the included catchments shown distinctly as outlines.
- The full set of existing and candidate protected areas, displayed with transparency or some other way of indicating where overlap occurs.
- A multi-panel plot showing how one candidate protected area network is compiled as an accumulation of the randomly selected candidate protected areas. Each step in the compilation does not need to be shown, but a representative selection of steps throughout the process should be displayed to give the reader an understanding of how networks were created. By including the total aggregate area of the protected area network at each displayed step, it will be easy for the reader to understand how the total area threshold was met and how overlap between candidate protected areas did not alter the candidate network area.
  - This will greatly clarify L.213-222. The discussion of overlap and its consequences (or lack thereof) was confusing without some frame of reference on which to base the statements. Showing where multiple candidate protected areas overlap (e.g., by displaying them with transparency in the figure) may help explain what is meant here. If this is made clear in the figure, then L.213-222 could be removed or greatly simplified. As long as the methods are clear that where candidate protected areas overlap only the aggregate area was considered (as indicated in the associated figure), that should be sufficient for the reader to understand what was done and why.

The above figures would likely benefit from use of color. Since PLoS ONE is online-only this will not result in extra charges.

Another helpful figure would be to depict functional connectivity. This is one of the two metrics employed in paper and yet the reader is left without a clear depiction of how functional connectivity actually works on the landscape. This also is relevant to L.414-416 in the Discussion. The paper states that a lack of functional connectivity during the last 20 years has led to division into sub-populations and heightened risk of extinction for caribou. This makes me wonder whether functional connectivity was overestimated in the models as it currently is hindered on the landscape. Without seeing any functional connectivity plots it is not possible to know how reasonable the model results for current and future functional connectivity might be. If the final functional connectivity map is considered protected information, since the caribou are an endangered population, a single iteration of the simulation could be shown as an example. This would at least give a sense of the model results. With this should be an indication of the location of the three caribou subpopulations references in L.269, as this will help the reader interpret the reasonableness of the functional connectivity results.

Modifications are also needed for Figure 4 to provide consistency and clarity with the results. Based on the text in L.474, a continuous color ramp of greyscale values was used, but only a single grey is shown in the figure legend. Also, as it currently stands the legend seems to indicate that the frequently selected candidate protected areas are shown in white, while in actuality these are the dark areas with a black outline. Changing this square to have a darker grey fill with a black outline would clarify this. Use of color, which does not cost extra in PLoS publications, would also be helpful here. Finally, the text refers to frequently selected candidate protected areas being adjacent to important caribou breeding habitats (L.337-338). Since these important breeding areas are referenced, they should be shown in Fig. 4 or Fig. 1.

Other methodological clarifications are also needed to allow the reader to assess the validity of the proposed methods:

- L.165-179. Prior to this point the reader has only been introduced to the existing protected areas and those proposed by MELCC, as shown in Fig. 1. It was not initially clear whether the approach to building protected areas networks outlined here would apply only to the proposed protected areas or to all lands in the study area. This should be clarified.
- L.183-184. Roads and trails are likely to be much smaller than a 250 x 250m raster pixel. Was any pixel overlapped by a road or trail considered to be a road or trail?
- L.184-185. The six disturbance types were given equal weight. Is there a reason to think that forestry activity and power line rights-of-way are as disruptive as roads or urban areas? In the absence of studies on caribou selection and avoidance equal weighting might be a reasonable assumption, but some support should be given from the literature for why caribou would be expected to respond strongly to things like forestry, rights-of-way and agriculture.
- L.185. Please give an example or two of what would be part of the “other” category.
- L.191-192. Is there any evidence that the median intactness outside of existing protected areas is functionally relevant for caribou movement? In other words, is having < 0.5% of a catchment intact really enough to allow for caribou movement? I have some doubts about this. Without a study of caribou movement with respect to intactness it will be difficult to determine what is possible, but at least it would be good to indicate why this is a reasonable assumption. Was any sort of sensitivity analysis conducted to see how this threshold affected the resulting networks?
- L.205-206. I do not understand what this means. Please clarify. For example, what does “protected area-level intactness criteria” mean? Is the point that intactness was only assessed at the catchment level, not for the resulting protected area? If so, this is not clear; I had to read it many times to come to this possible interpretation.
- L.233-234. “Catchment-level attributes were calculated using standard spatial data operations.” What does that mean? I do spatial analysis frequently, but I am uncertain what the authors consider “standard spatial data operations.” This needs clarification. If the point is simply to introduce the approaches of the following paragraph then delete this sentence, as it does not describe ‘standard measures.’ If it means something like calculating the mean or median across pixels, then state that instead.
- L.260. Consider updating to state that habitat generation was “to represent the current landscape and four landscapes for 2080 under future timber management and different climate change scenarios.” As currently stated, it is unclear how the current conditions map would differ from the scenario with no climate change impacts (L. 268) because it seems that only climate scenarios were changed. Based on the supplementary materials, however, it seems clear that timber management would lead to changes between the CC0 and current conditions map, even in the absence of climate effects. This should be indicated in the main text so those who do not read the supplement can still follow the basic approach.
- L.257. For readers not familiar with the approach of Bauduin et al. 2016 it would be helpful to give a brief summary of what the model does. For example (I made this up based on the information given here, not having read Bauduin et al. 2016), “The model simulates movements of individual caribou at an XX timescale with movement probability driven by habitat attraction/repulsion and an individual’s internal state [maybe an extra detail or two needed here]. Model outputs report the number of visits per landscape cell.” Such statements would give the reader the context to understand what was done and evaluate its relevance to the representation of functional connectivity. This could also be integrated with the content of L.269-272.
- No indication is given for what software were used to conduct the simulations, making replication difficult. This should be clarified. While the authors explain why the input caribou data cannot be shared due to endangered species considerations, it does seem feasible to share code or other materials that would allow others to apply their modeling approach.

The methods for generating future landscapes under climate change and management laid out in the supplementary materials also need some additional clarification. My primary concern is about how uncertainty was accounted for in generation of future landscapes. As best I can tell, future landscapes were only generated once for each climate scenario. However, at multiple steps in the process random selection and application of probabilities was applied (e.g., mature fir mortality due to spruce budworm, selection of fir stands outside of protection areas and conversion to other classes). If this is the case, the effects in future landscapes could be an artifact of these random selections and probabilities. Notably, the treatment of alpine habitat differed from that of fir habitat, as the former used fixed buffer distances, indicating that alpine habitat was always treated the same. This could result in different levels of variability across habitat classes. What impact did the random selection and use of probabilities have on outcomes? This paper generally does a good job of exploring many iterations of simulations to reflect potential variability, but here it is not clear that this was done. Some sort of sensitivity analysis or discussion would be nice.

Other aspects of the supplementary materials in which clarity is needed:

- Alpine tundra paragraph.
  - What were the buffered areas replaced with? I assume the classes along their borders. What happened if there was a mix of mature and regenerating fir stands along the boundary of alpine tundra?
- Mature fir stands and regenerating stands section.
  - Impact of climate change section
    - Here for the first time there is mention of an “other” category. Above only three habitats were mentioned as being used to generate habitat quality maps: alpine tundra, mature fir, and regenerating stands. How many habitat types were there actually? This needs to be made clear at the outset of the description. Also, what was lumped into the “other” category? The descriptions further in the supplement make it seem like it could contain fir trees from 30-50 years old, but this likely is very different functionally than if it consists of urban areas, agricultural areas, etc.
    - If potential habitat was determined to be lost, why were mortality probabilities set at < 1? It seems odd that this would happen. I understand doing this when habitat quality decreases, but not if it is lost. Without suitable habitat why should any fir forest be expected to remain, especially ≥ 50% of the time?
  - Impact of disturbances inside protected areas section
    - Good job identifying the potential for fire and windthrow to change with climate alteration as well as why these factors were not considered further. I would like to see more of this in the paper.
  - Impact of disturbances outside protected areas
    - What was done in the remaining 28% of forest outside of protected areas (if BFEC units cover 72% of the forest outside protected areas)? There is no description given for how habitat in these areas was treated.
    - It would be great to have a little more detail about how forestry plans for harvest are expected to increase the amount of old forest. This seems a bit counterintuitive if “the landscape is managed for timber production” as is stated above. I’m sure there’s a rationale for it, but for readers unfamiliar with the Québec context it would be great to explain briefly (e.g., by focusing timber harvest on currently regenerating stands and allowing older stands to grow unharvested…or whatever they are doing).

Another area where the manuscript should be strengthened is in the framing about protected areas and their designation, which is established at the beginning of the Introduction. The authors should be clearer about the choices they make and definitions they employ in their paper and should set these in the context of other possibilities with regards to conservation decisions. Protected area networks may be established for a variety of reasons. One important reason is maintenance of biodiversity, which is the case this paper focuses on (L.55-57). But others are more species-specific or culturally driven (e.g., IUCN categories III - VI), which may lead to other criteria for effectiveness. It is fine for the paper to focus on biodiversity protection as a primary goal of protected area networks, but this should be explicitly stated and complemented with at least a sentence or two recognizing other purposes for protected areas. The paper focuses strongly on effectiveness. Statements such as that “A high degree of ecological representativeness is a necessary condition for an effective protected areas network” (L.69-70), that “These effects would increase the effectiveness of a protected areas network” (L.79-80), and that the generated set of protected areas “may be close to ideal from an environmental or conservation point of view” (L.345) assume a certain criterion for determining an effective protected areas network, ignoring other potential goals. Thus, biodiversity maintenance is presented as if it were the only possible definition for effectiveness, rather than one of multiple possible definitions. I recommend a more explicit statement early in the Introduction (e.g., ‘we define an “effective” regional protected areas network as one that can sustain the region’s biodiversity into some reasonably foreseeable future”) and discussion of other possible definitions. Some of this is touched on in the Discussion (e.g., L. 347-359) but it would be better to set this up in the Introduction as well.

Other points to consider:

- L. 65-66. This assumes populations are stable or growing. The presence of species in ecological traps may artificially inflate representativeness, leading to assumption that “habitat requirements…will be satisfied within the protected areas network” when this is not actually the case. It might be nice to at least point out this possibility, even while acknowledging that this is nonetheless a typical assumption, as the paper does in L.68.
  - In fact, this is a strange statement given the following paragraph, which details why it is not the assumption is not necessarily reasonable. It seems like it would make more sense for the paragraph on representativeness to make the case for why representativeness matters for conservation (e.g., see second paragraph of Dietz et al. 2015 Biological Conservation and references therein).
- L.362. A citation is needed for this statement.
- L.366. Again, a citation is needed. In some cases, species thrive under habitat types resulting from human disturbances. It is a decision based on the sensitivity of focal species to human disturbance that should drive such decisions.
- L.376-377. This raises an important point about the potential to consider tradeoffs. This is not really done in the paper, however, as the focus is only on areas where both representativeness and functional connectivity are mutually high. One way this could be addressed is to make similar plots to Fig. 4 in the supplementary materials that are focused on just one of the indicators. Then the reader could better see where tradeoffs occur.

Other grammatical issues to address include:

- L.81. “among the main current drivers” reads a bit awkwardly. Suggest replacing with “a major driver.”
- L.90. Move comma within quotation marks.
- For clarity and better readability, I recommend moving “Because of…existing networks” (L.89-91) to before “The effectiveness of…” in L.84. and deleting “One important…functional connectivity” (L.91-92).
- L.111-121 are in present tense, L.122-134 in past tense. These should be consistent.
- L.152. “currently” is unnecessary. Delete.
- L.212. Remove extra “s” in “areass."
- L.252. Replace “or” with “of.”
- L. 256. Remove “by simulating.”
- L.269. Add an “s” to “subpopulation.”
- L.292. Remove “the” between “protected areas” and “most frequently selected.”
- L.374. Replace “ammeding” with “amending.”
- L.418. Remove “to” after “benefit.”
- L.427. Add “us” between “allows” and “to approximate.”
- L.465. Add an “s” to “represent.”
- Supplementary materials
  - Last sentence of first paragraph. Replace “caution” with “cautious.”
  - Alpine tundra paragraph.
    - Consider replacing “for the horizon 2090” with “by 2090.”
    - Add “using” between “…in Gaudry 2013)” and “interior buffering.”
    - Consider adding “soils” between “serpentine” and “(Sirois and Grandtner 1992)” and changing “is less subject” to “are less subject.”
  - Mature fir stands and regenerating stands section.
    - Impact of disturbances inside protected areas section
      - Consider replacing “turned” with “reclassified” in the second to last sentence
    - Impact of disturbances outside protected areas
      - Remove “for” in “Windthrow is similarly managed for.”
      - Replace “at” with “as” in “classed at mature.”
      - Insert “were” between “Once the landscapes” and “built with the.”
      - Consider rephrasing to something like “For the four climate change scenarios…the alpine tundra area was 100%, 59%, 39%, and 14%, respectively, compared to current conditions.
        - This will indicate what the percentages indicate (a comparison with current). Also, “remained at” indicates stasis while the numbers reported reflect decreases in area.
        - Similar phrasing should be used for changes in area of mature fir stands and regenerating stands.
      - Consider deleting “fir stands area increased” as this seems to still be explaining why old stand area increased.
      - Consider adding “regenerating” after “However,” as this sentence seems to be just about regenerating stands since old stands mostly increased.
